# The hepatopancreas microbiome of velvet crab, *Necora puber*

**DOI:** 10.1111/1758-2229.70014

**Published:** 2024-10-01

**Authors:** Signe Martin, Cindy Smith, Kelly Stewart, William Barr, Deborah Cheslett, Ian O'Connor, Fiona Swords, Umer Zeeshan Ijaz, Katie O'Dwyer

**Affiliations:** ^1^ Marine and Freshwater Research Centre Atlantic Technological University Galway Ireland; ^2^ James Watt School of Engineering University of Glasgow Glasgow UK; ^3^ Marine Institute Oranmore Ireland

## Abstract

Crustaceans are a valuable resource globally, both ecologically and economically, and investigations into their health are becoming increasingly important as exploitation rises. The microbiome plays a crucial role in crustacean immunity, and understanding its composition and structure can provide insights into the health of an organism and its interactions with various factors. In this study, we investigated the hepatopancreas microbiome of the velvet swimming crab, *Necora puber*, and compared its composition and structure with several study factors, including two different sampling points and infection with a paramyxid parasite, *Paramarteilia canceri*. To our knowledge, we provide the first description of a velvet crab microbiome, highlighting the dominance of a single microorganism, *Candidatus hepatoplasma*. We identified variations in microbiome composition between sampling points and discussed the possible processes affecting microbiome assembly. We also outline a core microbiome for the velvet crab hepatopancreas, consisting of 12 core phyla. Our study adds to the growing literature on crustacean microbiomes and provides a baseline for future investigations into the velvet crab microbiome and the health of this crustacean species.

## Graphical abstract

We provide the first description of the hepatopancreas microbiome of the velvet crab, *Necora puber*, identifying a microbial community dominated by *Candidatus hepatoplasma* and the presence of a core microbiome. We also investigate the variation explained by our study factors: crab sex, parasite infection status, and two different sampling time points.

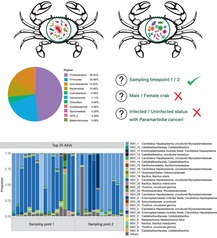

## INTRODUCTION

Both wild and cultured crustaceans are a valuable resource globally, making up 23% of the value of the global trade of aquatic animal products in 2022 (FAO, 2024). Consumption of crustaceans has increased between 1961 and 2022, compared to a decrease in finfish (FAO, 2024), and the health of these animals is important for continued sustainable exploitation, as demand for these organisms continues to grow (Boenish et al., 2022; Stentiford et al., 2012). The microbiome of an organism plays an important role in maintaining health, assisting in functions such as digestion, immunity, and protection from pathogens (Hoffmann et al., 2016; Holt et al., 2021). A clear understanding of an organism's microbiome provides opportunities for a broad range of applications, including treatments to improve the health of aquaculture species (Berg et al., 2020; Rajeev et al., 2021). Changes to the microbial community composition can contribute to the development of disease (Rajeev et al., 2021; Sharpton & Gaulke, 2015), and in particular, reduced bacterial diversity in the microbiome can lead to reduced health and disease development (Cornejo‐Granados et al., 2017; Holt et al., 2021). Microbiome composition may vary due to a range of factors, although, typically a ‘core’ microbiome can be identified which is composed of microbes that endure, or remain stable, despite varying conditions (Kokou et al., 2019) and can be crucial in maintaining health (Perlman et al., 2022).

Factors which may alter microbiome composition in crustaceans include internal factors such as moulting, as well as external factors such as temperature changes (Apine et al., 2021; Zhang et al., 2021). Diseases, pathogens, and parasites can also alter the microbiome, leading to compromised host health (Ding et al., 2017). A range of microbiomes have been studied in crustaceans, including those found in the Chinese mitten crab *Eriocheir sinensis* hepatopancreas (Yu et al., 2021), mud crab *Scylla serrata* intestine (Apine et al., 2021), edible crab *Cancer pagurus* carapace (Bergen et al., 2022), Pacific white leg shrimp *Litopenaeus vannamei* hepatopancreas and intestine (Cornejo‐Granados et al., 2017), and signal crayfish *Pacifastacus leniusculus* in a range of tissues (Dragičević et al., 2021). The hepatopancreas (or midintestinal gland) is an organ of the crustacean digestive system. It produces digestive enzymes, absorbs nutrients (Ceccaldi, 1989; Rőszer, 2014), and has a role in crab growth, moulting, and reproduction (Jiang et al., 2009; Mykles, 2011; Wang et al., 2014). A range of studies have examined the hepatopancreas microbiome in several crustaceans, and changes in composition were recorded due to a range of factors including disease and/or pathogen infection (López‐Carvallo et al., 2022; Shen et al., 2017), wild versus cultured individuals (Cornejo‐Granados et al., 2017), geographic range (Dragičević et al., 2021), and moulting cycle (Zhang et al., 2021). In one study on the isopod species complex, *Jaera albifrons*, Wenzel et al. (2018) reported that a significant amount of the variance in microbiome composition was explained by host sex, and differences in sampling location and time.

Microbiome investigations can shed further light on the processes involved in the development of infections and disease in crustaceans. The hepatopancreas can harbour parasite infections, such as the protozoan microparasite *Paramarteilia canceri* in velvet crab, *Necora puber*, (Martin et al., 2024), which may contribute to the mortality of this valuable commercial crustacean (Collins et al., 2022). Currently, there are no descriptions of microbiomes from velvet crab, and for sustainable management of this crab, investigations into the microbiome may be a useful tool for clarifying the health status of velvet crab populations and stocks.

Exploring microbiome composition can also elucidate the functions of different microbes in crustaceans (Zeng et al., 2016) and the processes and mechanisms that affect community composition and structure (Burns et al., 2015; Zhou et al., 2013) which may be stochastic or deterministic (Zhou & Ning, 2017). Niche theory hypothesises that deterministic processes affect community composition, including predation, dispersal limitation, environmental factors, and species traits, while neutral theory hypothesises that stochastic processes of birth, death, and others such as speciation, affect the community structure (Zhou & Ning, 2017). It is now more accepted that these processes co‐occur in their impact on microbial community structure (Zhou & Ning, 2017). However, these mechanisms and their impacts on the microbiome still require more research to be fully understood.

Here we provide a first description of the hepatopancreas microbiome of the velvet crab, *N. puber*. We identify a core microbiome using abundance‐occupancy distributions and examine the microbes which are likely to be host‐selected or selected by dispersal limitation using a neutral model (Burns et al., 2015; Shade & Stopnisek, 2019). Host selected refers to taxa occurring more often than predicted by the model and are hypothesized to be selected for or maintained by the host, while taxa occurring less frequently than predicted are more likely to be dispersal limited (the neutral model suggests this as organisms lost will be replaced by individuals outside the local community) (Burns et al., 2015). We also compare microbiome composition across several different factors including crab size, crab sex, two different sampling time points, and infection status (with the parasite *P. canceri*). In addition, several microbes are investigated in greater detail to shed light on the possible implications of their presence.

## EXPERIMENTAL PROCEDURES

### Sampling/dissection

A total of 30 velvet crabs were collected live from a fisher fishing with crab pots in Galway Bay at two different time points, one collection in March 2021 (*n* = 16), and one in June 2021 (*n* = 12), representing Sampling point 1 and Sampling point 2. Crabs were transported back to the laboratory in buckets and placed on ice 30 min prior to dissection to anaesthetise them (Collins et al., 2022). All crabs were dissected on the day of collection. All dissecting tools were washed and rinsed in 99% ethanol between each crab. The sex of the crabs was determined based on the shape of the abdomen, which is v‐shaped in males and more rounded in females with a covering of setae (Norman, 1989), and sex was also confirmed internally during dissection. The size of individual crabs was recorded as the carapace width at the widest part of the carapace (Hearn, 2001), and to the nearest 0.5 cm using a ruler. Next, the legs and chelae were removed, followed by opening of the carapace using scissors. A sample of hepatopancreas was removed using forceps and placed into a sterile Eppendorf tube which was frozen at −80°C until DNA extractions were performed.

### Parasite detection

*Paramarteilia canceri* infections were confirmed as part of a previous study (Martin et al., 2024). The QIAamp DNA mini kit was used to extract DNA from velvet crab hepatopancreas samples using a QIAcube robot as per the manufacturer's instructions (QIAGEN). PCR screening for *P. canceri* was carried out using the primers PMart_18S_For and PMart_58S_Rev (of Collins et al., 2022; Sigma‐Aldrich). These primers amplify the ITS1 regions of the 18S rRNA gene. The reaction mixture was composed of 1 μL of extracted DNA, 1x GoTaq buffer, 2.5 mM MgCl_2_, 0.4 mM dNTPs, 0.5 μM of each primer, and 0.5 U GoTaq Flexi polymerase (Promega) for a final volume of 20 μL. PCR cycle conditions consisted of a 5‐min denaturation at 95°C, 35 amplification cycles of 95°C for 1 min, 62°C for 1 min and 72°C for 1 min, finishing with a 10 min extension at 72°C and storage at 4°C (Collins et al., 2022). PCR products were visualized on 1.2% agarose gels stained with ethidium bromide (Promega). Amplicons of approximately 650 bp in length were produced.

### Microbiome DNA analysis

DNA was extracted from the velvet crab hepatopancreas using the QIAamp DNA mini kit from Qiagen. The primers F27 (AGAGTTTGATCMTGGCTCAG) (Yuan et al., 2012) and R338 (GCTGCCTCCCGTAGGAGT) (Amann et al., 1990) were used to amplify the V1/V2 regions of the 16S rRNA gene (Salter et al., 2014). There were 15 forward and 12 reverse primers with a universal Illumina adapter sequence (Eurofins), an index, and some with added ‘heterogeneity spacers’ adapted from Fadrosh et al. (2014) for added flexibility (see Table S1 for more detail). Primer conditions were optimized and the primer combinations that were chosen for each sample are shown in Table S1. A selection of six negative controls was also sent for sequencing, these included four blank DNA extraction samples as a control for the DNA extraction set and two blank PCR products as a control for the PCR reaction.

A 20 μL reaction mixture was composed of 2 μL of DNA template, 0.2 mM dNTPs, 1.5 mM MgCl_2_, 0.3 μM of each primer, 1x GoTaq buffer, and 0.5 U GoTaq Flexi polymerase (Promega). PCR cycling conditions were 95°C for 5 min, 30 cycles of 95°C for 30 s, 57°C for 30 s and 72°C for 30 s, with a final extension step of 72°C for 10 min (Collins et al., 2022). PCR products were visualized on a 1.2% agarose gel with a GeneRuler 100 bp ladder. Amplicons of approximately 450 bp were produced. For each sample with a positive band in the gel, two to three individual PCR products were amplified and combined before purification to ensure the DNA concentration was at the required minimum of 5 ng/μL. For purification, the NucleoSpin gel and PCR purification kit were used, and each sample was eluted with buffer to produce a final volume of 25 μL. Samples were quantified using a Nanodrop spectrophotometer.

Samples were sent for sequencing at Eurofins Genomics Germany, where amplicons were sequenced using the Illumina MiSeq platform.

### Bioinformatics

Using Fadrosh et al. (2014), the resulting reads obtained from the sequencing centre had the following format: [Multiplexing Barcode] + [Heterogeneity Spacer] + [Primer Sequence]. To remove heterogeneity spacers and match primer sequences, we used CutAdapt (Martin, 2011). Abundance tables were then generated by constructing amplicon sequencing variants (ASVs) in the QIIME2 workflow (Bolyen et al., 2019) using the DADA2 denoising algorithm (Callahan et al., 2016). The commands are given at: https://github.com/umerijaz/tutorials/blob/master/qiime2_tutorial.md. The results provided an *n* = 36 × *P* = 2724 ASVs abundance table. The summary statistics of sample‐wise read distribution mapping to these ASVs is: Minimum: 9238; 1st Quartile: 69,139; Median: 80,544; Mean: 78,005; 3rd Quartile: 89,170; Maximum: 111,533. We then classified the ASVs using the recent SILVA SSU Ref NR database release v.138 (Quast et al., 2012) and combined the taxonomic information with the abundance table to generate a BIOM file. The rooted phylogenetic tree was generated using FastTree2 (Price et al., 2010) within the QIIME2 framework to remove phylogenetically ambiguous alignments, along with the BIOM file.

### Statistical analyses

All the subsequent downstream analyses were done in R (R Core Team, 2022) and RStudio version 4.1.3 (RStudio Team, 2022). As a pre‐processing step, we removed typical contaminants such as mitochondria and chloroplasts, as well as any ASVs that were unassigned at all levels, as per recommendations given at https://docs.qiime2.org/2022.8/tutorials/filtering/. We further used R's *decontam* package (Davis et al., 2018) to identify and remove contaminants using blank control samples, and by employing the ‘Frequency Method’ in the package. Afterwards, R's *vegan* package (Oksanen et al., 2008) was used for alpha and beta diversity analyses. For alpha diversity, we used: (i) Pielou's evenness; and (ii) rarefied richness. For the alpha diversity comparison between multiple categories, we have used ANOVA using the aov() function in R. Beta diversity was calculated using two different distance measures: (i) Bray–Curtis distance on the ASV abundance table to visualize the compositional changes; and (ii) Unweighted UniFrac distance estimated using R's *phyloseq* package (McMurdie & Holmes, 2013) to examine phylogenetic differences between samples. To visualize the samples in reduced dimensions using the Bray–Curtis and unweighted UniFrac distances, Principal Coordinates Analysis (PCoA) was used. Additionally, the R *vegan* package was also used to perform permutational multivariate analysis of variance (PERMANOVA) to examine variability in the microbial community structures. We have performed PERMANOVA only on covariates selected by the ‘redundancy analysis with forward selection’ strategy (again using the R *vegan* package) as used previously (Vass et al., 2020).

To identify the core microbiome, we used the approach discussed in Shade and Stopnisek (2019). The approach first ranks the ASVs using two metrics: site‐specific occupancy (samples were grouped by a combination of the study factors: sampling point, sex, and infection); and replicate consistency (whether the ASVs are consistent across replicates). After ranking the ASVs, the subset of core taxa is constructed incrementally by adding highly prevalent to lowly prevalent ASVs and then quantifying the contribution of the core subsets to beta diversity using the Bray–Curtis distance in the equation.

$$C=1-\frac{BC_{\text{core}}}{BC_{\mathrm{all}}}.$$

Two approaches are specified (Shade & Stopnisek, 2019) to decide at what threshold the core subset construction stops: (a) where the addition of an ASV does not cause more than a 2% increase in the explanatory value by Bray–Curtis distance; and (b) an ‘elbow’ approach where first‐order differences are calculated by partitioning the curve in two parts, and calculating the difference in the average rates of change for both of these parts. The point at which this difference is maximized is the elbow point. Approach (b) is very stringent and therefore approach (a) was used as recommended by the original authors (Shade & Stopnisek, 2019). Independently, a neutral model (Burns et al., 2015) was fitted to the ‘S’ shaped abundance‐occupancy distributions to provide information on the ASVs that are likely selected by the environment. These are obtained as those that fall outside the 95% confidence interval of the fitted model and are inferred to be deterministically assembled, rather than neutrally selected, with those points that are above the fitted model values indicating those selected by the host environment, and those points below the model showing those that are dispersal limited. The taxonomy tree of the core microbiome across different occupancies and the collated core (all occupancies added together) were drawn using R's metacoder package (Foster et al., 2017). Given three study factors with two levels each, there were eight possible occupancies, however, there were no samples representing one of these (Sampling point 2/uninfected/male). The seven different occupancies were representative of two sampling points SP [1/2], infection status with *P. canceri* I [Yes/No], and sex S [Male/Female], and the seven are *SP:1 I:N S:F*; *SP:1 I:N S:M*; *SP:1 I:Y S:F*; *SP:1 I:Y S:M*; *SP:2 I:N S:F*; *SP:2 I:Y S:F*; *SP:2 I:Y S:M*.

To find genera (ASVs collated at genus level based on SILVA SSU Ref NR database release v.138) that differed significantly between the two sampling points we used the DESeq2 package (Love et al., 2014) with the adjusted *p*‐value significance cut‐off of 0.05 and log2 fold change cut‐off of 2. The function uses negative binomial generalized linear modelling to obtain Maximum Likelihood estimates for genera undergoing log fold changes between two conditions (sampling point in this case). Bayesian shrinkage is then applied to obtain shrunken log fold changes, subsequently employing the Wald test for obtaining measures of significance. TSS + CLR (Total Sum Scaling followed by Centralized Log Ratio) normalized abundances were used to visualize the discriminatory genera.

To find the relationship between microbial communities and all sources of variation, we have used a Generalized Linear Latent Variable Model (GLLVM) (Niku et al., 2019) which extends the basic generalized linear model that regresses the mean abundances $\mu_{\mathrm{ij}}$ (for i‐th sample and j‐th microbe) against all sources of variation $x_{i}$ by incorporating latent variables $u_{i}$ as $g\left(\mu_{\mathrm{ij}}\right)=\eta_{\mathrm{ij}}=\alpha_{i}+\beta_{0j}+x_{i}^{T}\beta_{j}+u_{i}^{T}\theta_{j}$, where $\beta_{j}$ are the microbe‐specific coefficients associated with individual covariates. Once estimated, a 95% confidence interval of these coefficients, whether positive or negative and not crossing 0, gives directionality (e.g., the interpretation that an increase in a covariate causes an increase in the abundance of a microbe). $\theta_{j}$ are the corresponding coefficients associated with latent variables. $\beta_{0j}$ are microbe‐specific intercepts, while $\alpha_{i}$ are optional sample effects which can either be chosen as fixed effects or random effects. To model the distribution of individual microbes, we used a negative binomial distribution with an additional dispersion parameter and used log() as a link function. Additionally, the approximation to the log‐likelihood is done through variational approximation (VA) with the final sets of parameters in glvmm() function being family = ‘negative.binomial’, method = ‘VA’, and control.start = list(n.init = 7, jitter.var = 0.1), these parameters resulted in the algorithm achieving convergence.

### Decontamination

In the pre‐processing step, decontamination removed 18 contaminants across the 30 samples. The 16S rRNA sequencing generated a total of 2724 ASVs after this pre‐processing step and unassigned ASVs made up 1.62% of these. As an additional decontamination step, unassigned ASVs and 174 ASVs identified as contaminants using the blank controls and R's *decontam* package (Davis et al., 2018) were removed. Further, two samples were removed from the dataset for all statistical analyses after investigating sample composition and finding that these two samples had a very similar composition to the negative control and were suspected to be contaminated.

## RESULTS

### Data summary

Samples of hepatopancreas from a final total of 28 velvet crabs were analysed to examine the composition of the microbiome. These samples consisted of 13 males, 15 females, 16 from Sampling point 1, and 12 from Sampling point 2, and *P. canceri* infection was detected in 19 samples (9 samples uninfected).

After decontamination, there were 2506 remaining ASVs which were assigned to 26 phyla, 65 classes, 172 orders, 263 families, 436 genera, and 254 species.

### Microbiota composition

**FIGURE 1:**
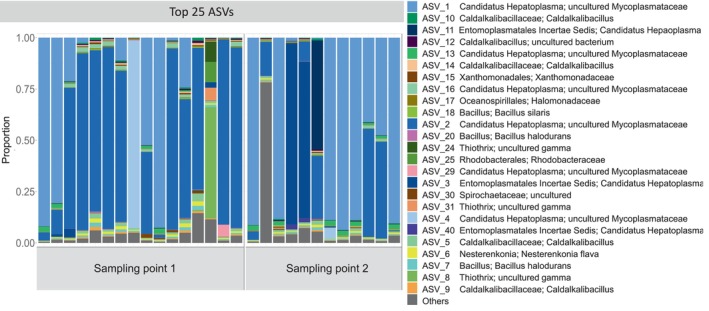
The top 25 most relatively abundant ASVs in each of the 28 hepatopancreas samples, were identified at two different sampling points. ‘Others’ = all other ASVs not included in the top 25.

The top 25 most abundant ASVs of the velvet crab hepatopancreas microbiome were identified (Figure 1) and the community was dominated by *Candidatus hepatoplasma*, which accounted for the majority of microbiota in 26 of the 28 samples, with 94% as the highest relative abundance from a single *C. hepatoplasma* ASV in one sample. Six of the nine different variants of *C. hepatoplasma* were further defined as uncultured Mycoplasmataceae. The dominance of *C. hepatoplasma* variants was evident across both sampling points (Figure 1). The two remaining samples consisted of one with a large proportion of the genus *Thiothrix* (54% relative abundance), and another with a large proportion of ‘others’ or undefined (Figure 1).

### Microbiome variation

To examine factors potentially driving variation in the microbial community we carried out a PERMANOVA analysis, which identified the sampling point as the only significant factor (Table 1) using both Bray–Curtis distance (*R* ^2^ = 0.14, *p* = 0.013) and Unweighted‐UniFrac distance (*R* ^2^ = 0.06, *p* = 0.005, see Table S2).

**TABLE 1:** PERMANOVA results based on the Bray–Curtis dissimilarity matrix used to assess the difference in ASVs across four different study factors.

	Covariates	Degrees of freedom	Sum of squares	*R* ^2^	*F*	*p*‐value
Bray–Curtis distance	Sampling point	1	1.06	0.14	4.13	**0.013**
	Infection	1	0.19	0.03	0.75	0.530
	Crab sex	1	0.14	0.02	0.56	0.666
	Crab size	1	0.13	0.02	0.52	0.726
	Residual	23	5.89	0.79		
	Total	27	7.42	1.00		

*Note*: Significant value indicated in bold.

**FIGURE 2:**
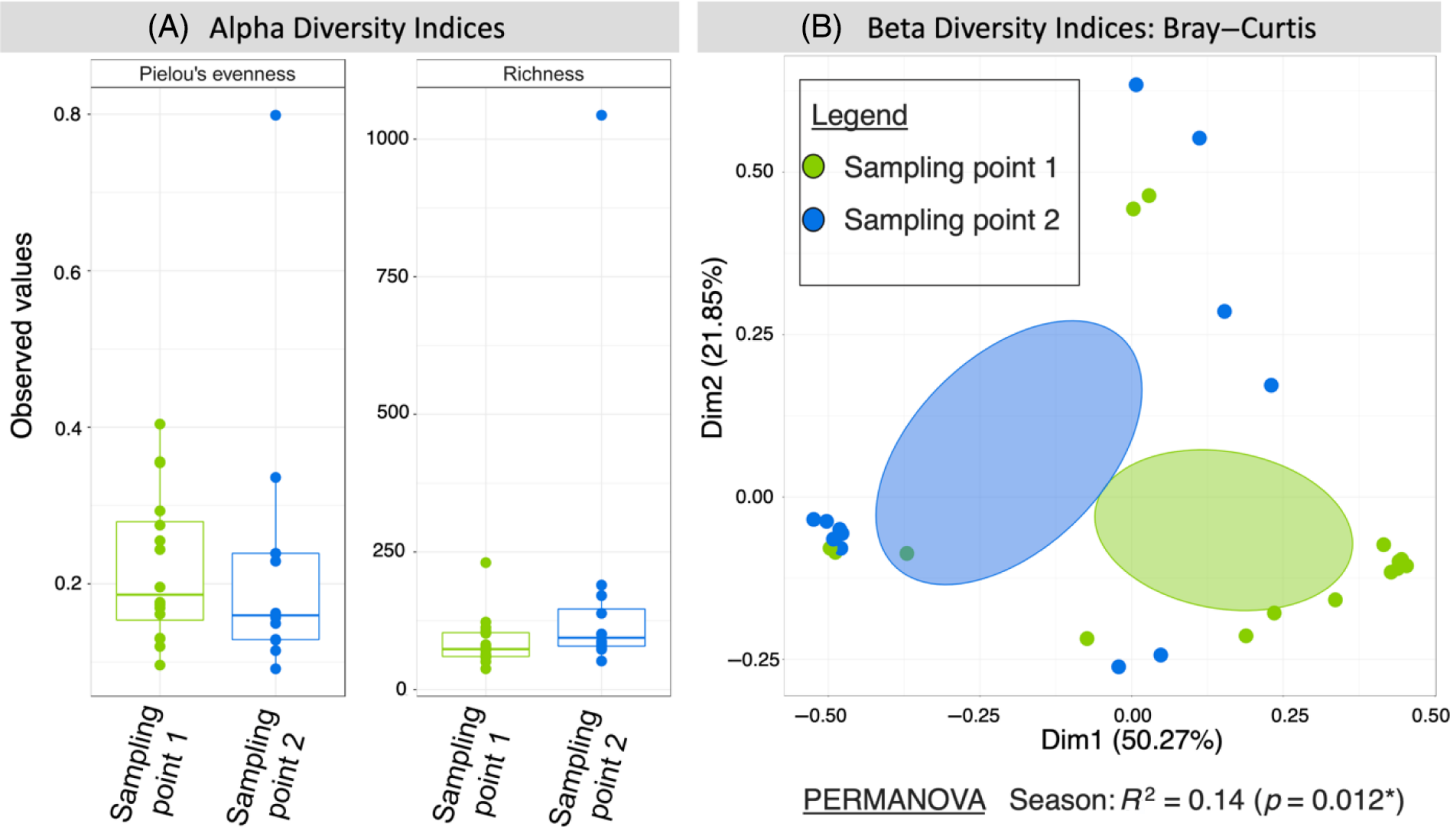
Microbial diversity of ASVs in the hepatopancreas of velvet crab. (A) Alpha diversity indices showing Pielou's evenness, and rarefied richness. Boxplot legend: Box = inter‐quartile range (25% and 75% quartile), line = mean values, * = significant result. (B) Beta diversity index showing Bray–Curtis dissimilarity matrix results with PERMANOVA *R* ^2^ and *p* values provided below the plot. The ellipses represent the 95% confidence interval of the standard errors of the points of a given group (Sampling point 1 or 2).

As the sampling point was the only factor identified as significant in explaining differences in ASVs in velvet crab hepatopancreas microbiome diversity, analyses examining both alpha and beta diversity were grouped by Sampling points 1 (*n* = 16) and 2 (*n* = 12). In assessing alpha diversity, the ASV rarefied richness of the samples ranged from 37.85 to 228.67 ASVs, except for one sample with a richness of 1042.57 ASVs. The average richness of the microbial community, excluding this one outlier, was 93.1 ± 44.43 ASVs. Pielou's evenness produced a median value of 0.19 and the average evenness was 0.23 (±0.141). There was no significant difference between sampling points for either richness or evenness (*p* = 0.17, *p* = 0.86) (Figure 2A). The low evenness in ASVs here is further supported by the dominance of few ASVs (*C. hepatoplasma*) in the top 25 most abundant ASVs (Figure 1). Beta diversity demonstrated clustering of the two different sampling points (Figure 2B), and a significant difference was found between them in both the Bray–Curtis distance (*R* ^2^ = 0.14, *p* = 0.012) and the Unweighted UniFrac distance (*R* ^2^ = 0.06, *p* = 0.006, see Figure S1).

**FIGURE 3:**
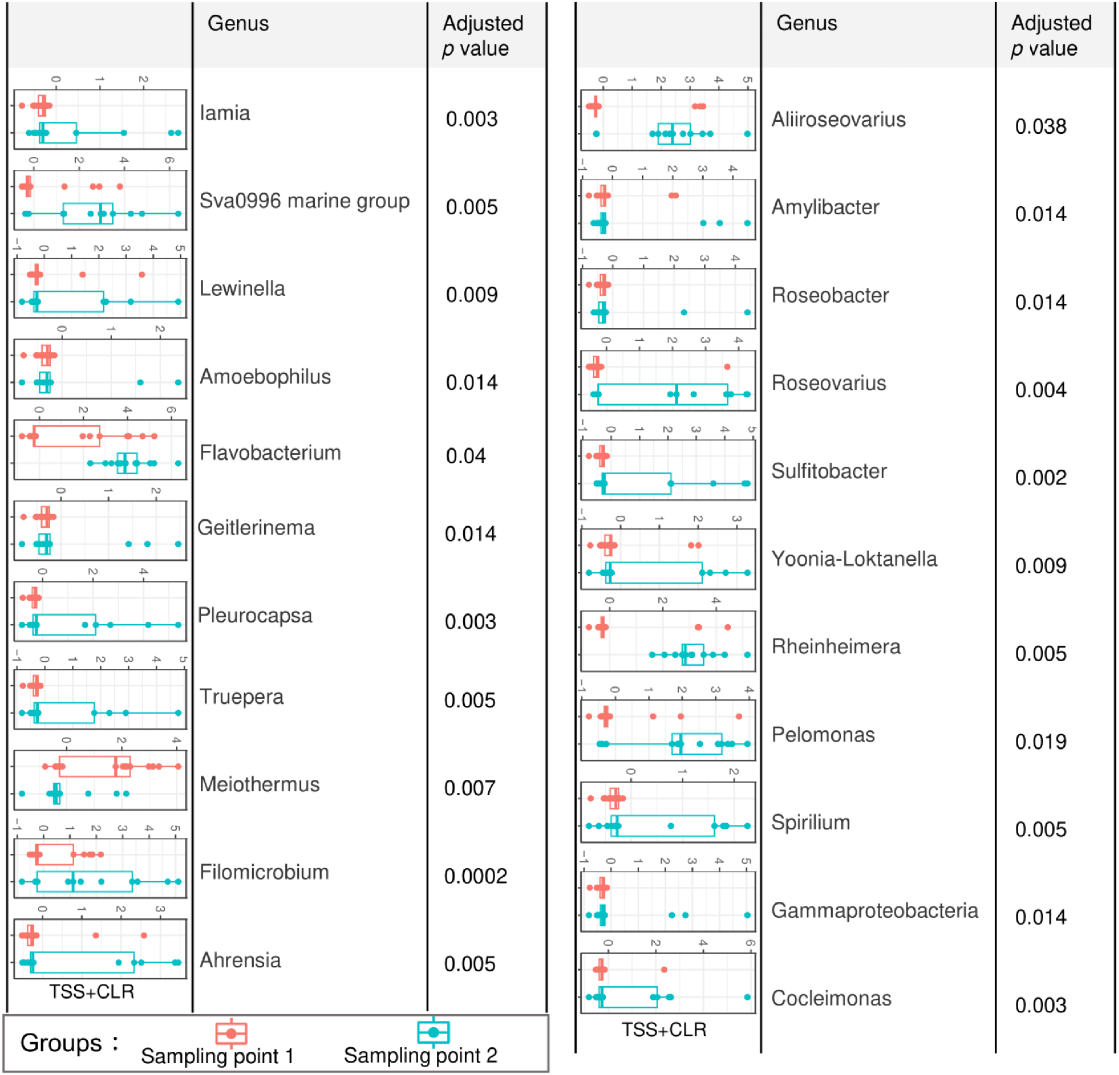
Differential analysis of relative abundance shows genera that were significantly different between sampling points one and two, with adjusted *p*‐values with a significance cut‐off of 0.05 and log2 fold change cut‐off of 2 after employing the Wald statistics. Boxplot legend: box = inter‐quartile range (25% and 75% quartile), line = mean values.

While the majority of velvet crab hepatopancreas samples were dominated by *C. hepatoplasma* ASVs some variation in ASVs was detected between samples collected at the two different sampling points. To explore this variation a differential analysis was carried out. Through this analysis, we identified the specific ASVs which significantly differed between Sampling points 1 and 2 (Figure 3). except for *Meiothermus*, all other ASVs were significantly more abundant in Sampling point 2 (Figure 3).

The results of the GLLVM revealed which microbes are positively and negatively associated with the sampling point. The sampling point was the only factor which explained variation in the microbiome and Table 2 shows the top five ASVs (at genus level) most positively and negatively associated with Sampling point 1. Positively associated ASVs were more often found in Sampling point 1, and negatively associated ASVs were more often found in Sampling point 2. The ASVs identified have previously been isolated from a range of different sources. Six ASVs were previously isolated from environmental samples, including air, water, and sediment. Six ASVs were isolated from animal sources previously, four of which were from invertebrates, with one of the four (*Bdellovibrio*) isolated from a crustacean. One ASV was previously isolated from a plant source. For all these ASVs, a range of different functions have been implied in the literature and these are summarized in Table 2. Some of the functions identified include use as a probiotic, degradation of organic compounds, and control of other bacteria. Conversely, some of the less studied ASVs do not have clear implications or functions already identified. All other covariates, crab sex, crab size, and infection status (infected/uninfected with the parasite *P. canceri*) were also analysed for differences in the microbiota associated with each of them and the results of the analysis can be found in Figure S2, i–ix and the literature survey in Table S3. Of the ASVs identified as the top five most positively or negatively associated with these other covariates, four had been associated with crustaceans before, and two with disease (Table S3).

**TABLE 2:** Literature survey of the top five most positively (blue) and negatively (red) associated ASVs with the covariate sampling point.

Genus	Literature review details	Isolation source	Reference
*Candidatus Moranbacteria*	May degrade chitin and carry out fermentation that forms acetate. Found in contaminated groundwater and radiation and chemolithotrophic environments.	Environment (water)	(Nayak et al., 2021, Van Der Waals et al., 2018, Vigneron et al., 2020)
*Lentilitoribacter*	Aerobic bacteria. Core gut microbiota of tropical gar *Atractosteus tropicus*.	Animal (fish)	(Méndez‐Pérez et al., 2019, Park et al., 2013)
*Flavobacteriales*, NS9marine_ group	Degrades complex organic compounds.	Environment (water)	(Yeh & Fuhrman, 2022)
*Brevibacillus*	Thermophilic bacterium. Used as a probiotic in human health, aquaculture, and livestock.	Environment (sediment, water), animal (invertebrate)	(Wang et al., 2021)
*Iamia*	Aerobic bacteria. Isolated from sea cucumber and marine sponges. The implication is unclear.	Animal (invertebrate)	(Kurahashi et al., 2009, Khan et al., 2012)
*Effusibacillus*	Thermo‐ and acidophilic bacteria. Has been isolated from farm soil and lake sediment.	Environment (sediment)	(Konishi et al., 2021, Wang, Berdy, et al., 2020)
*Tepidimonas*	Chemolithoheterotrophic and slightly thermophilic denitrifying bacteria. Associated with pancreatic cancer tumour tissue. Found in fish gut microbiota.	Animal (fish)	(Jeong et al., 2020)
*Janibacter*	Halophilic and thermophilic. Reported to cause bacteraemia in humans and has been isolated from various marine environmental sources. Degrades aromatic hydrocarbons.	Animal (invertebrate), environment (air, sediment)	(Castilla et al., 2017, Elsayed & Zhang, 2005, Lim et al., 2017)
*Xylella*	Aerobic bacteria that are a major pathogen in plants.	Plant	(Saddler & Bradbury, 2015, Sicard et al., 2018)
*Bdellovibrio*	Predatory bacteria, possible use to control vibriosis in shrimp mariculture. Halophilic bacteria isolated from many marine sources.	Environment (water), animal (crustacean)	(Wen et al., 2009)

*Note*: Positively associated genera were most associated with Sampling point 1, while negatively associated genera were most associated with Sampling point 2.

### Core microbiome

**FIGURE 4:**
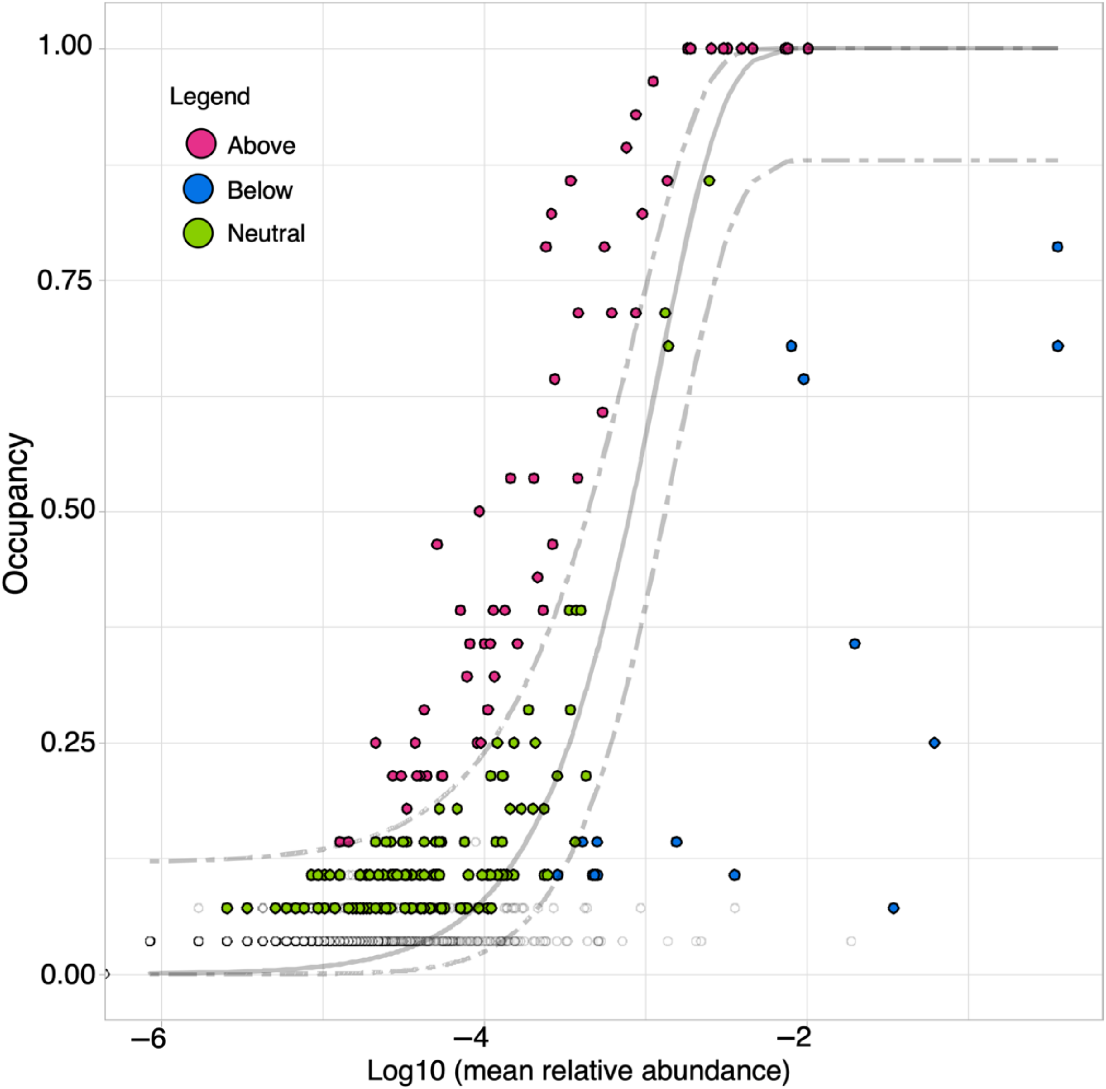
Core ASVs of the velvet crab hepatopancreas microbiome. Abundance‐occupancy distribution shows which ASVs are neutrally selected (green), and which are likely to be deterministically assembled, those above the model were identified as host‐selected (pink) and those below the model as dispersal‐limited (blue).

A core microbiome was identified for the hepatopancreas of velvet crab examined here and consisted of 12 core phyla (Figure S3). The most abundant phyla were Pseudomonadota (45.54%), Bacillota (24.41%), Actinomycetota (14.06%), and Bacteroidota 10.80%. All other phyla made up less than 10% of the microbiome (Figure S3). When considering ASVs rather than phyla, a total of 210 core ASVs were identified. Of these ASVs, 137 were indicated as neutral, 58 as likely to be host selected, and 15 indicated as dispersal limited selected (Figure 4). Some of the core ASVs identified as host selected belong to the genera, *Bacillus*, *Caldalkalibacillus*, as well as *C. hepatoplasma* and *Thiothrix* which were in the most abundant ASVs (Figure 1).

Since we have used an occupancy model in the core microbiome calculation, further investigating the core ASVs through taxonomic coverage trees (Figure S4, i–viii) at site‐specific occupancies (all combinations of study factors: crab sex, infection status, and sampling point and then the collated core) highlighted that some occupancies have a distinct core. These distinctions were obvious with the Sampling point 1/infected/female occupancy (Figure S4, iv) which had many underrepresented branches or lower abundance (represented by grey colour in the taxonomic coverage trees), and a smaller number of distinct taxa (represented by node width in the taxonomic coverage trees). The occupancy Sampling point 1/infected/female (Figure S4, ii) also had many underrepresented branches, while the occupancy Sampling point 2/infected/female (Figure S4, vii) stood out for having well‐represented taxa (wider nodes and darker colours in the taxonomic coverage trees). Differences could also be seen when focusing on specific taxa. This was most obvious with Illumobacter, Kilioniellales, Rhodobacterales, and Flavobacteriales which were better represented in the occupancy Sampling point 2/infected/male (Figure S4, viii).

## DISCUSSION

The hepatopancreas microbiome of the velvet crab, *N. puber*, was examined and a total of 2506 sequence variants (ASVs) were identified as associated with the hepatopancreas. Crabs sampled in this study were collected across two different sampling points and their microbiomes were found to differ significantly between sampling points. Our tests for microbiome variation in relation to crab sex, infection status with the protozoan parasite *P. canceri* and for crabs of different sizes yielded no significant differences. We established taxa composition, analysed microbial diversity, and investigated the presence of a core microbiome in the hepatopancreas of velvet crabs.

Investigations of the composition of each sample, and identification of the top 25 most abundant ASVs revealed that almost all samples were dominated by *C. hepatoplasma*, which was previously found to dominate the hepatopancreas microbiome of the American lobster, *Homarus americanus* (Schaubeck et al., 2023). *Candidatus hepatoplasma* is a colonizing symbiont bacterium (Wang et al., 2004) which has also been identified in the core gut microbiome of the mud crab, *Scylla paramamosain*, and was in the top 15 genera by relative abundance in the same species (Jiang et al., 2023). The same bacterium was previously observed to improve the survival rate of nutritionally stressed isopods (Fraune & Zimmer, 2008) and has a possible role in food degradation (Bredon et al., 2021). The dominance of *C. hepatoplasma* in the microbiome here indicates it may have an important function in the hepatopancreas, such as in food degradation which has been suggested previously (Bredon et al., 2021). Only two samples were not dominated by *C. hepatoplasma*, one which had a high proportion of undefined ASVs and one with a high proportion of *Thiothrix*, a genus identified as an ectosymbiont of the caecum of the echinoderm *Echinocardium cordatum* and which may play a role in digestion (Brigmon & De Ridder, 1998).

In investigating variation in the microbiome composition with the study factors, the sampling point was the only factor for which significant differences in the hepatopancreas microbiome were identified. Of the ASVs which significantly differed between the two sampling points, all except one ASV were more abundant in Sampling point 2. Differences in microbiome composition are commonly seen with changes in time, even within a 24‐h period, which has been shown in Chinese mitten crab (*E. sinensis*) gut microbiota (Yu et al., 2021). Here, Sampling point 1 was in March and Sampling point 2 was in June of the same year. The variation could be explained by environmental factors such as a temperature change, which has been shown in mud crab, *S. serrata*, intestine microbiome, where decreased richness in microbiota was reported with increasing temperature over an 8°C difference (Apine et al., 2021). In this study, the average temperature increase was 7.7°C between sampling points, In March 2021 average sea temperature was 7.7°C and doubled by June 2021 at 15.4°C (Digital Ocean, 2021). Gonad development has also been shown to affect microbiome composition, with a study on the shrimp *Neocaridina denticulata*, finding that gonad development significantly affected microbiome richness and diversity in the gut, but that any changes detected were insignificant in the hepatopancreas (Cheung et al., 2015). These results suggest that the hepatopancreas microbiome may be relatively stable. Nonetheless, for the velvet crab examined here March and June are both periods indicated for gonad development (Bakir & Healy, 1995) and therefore this may not be an influential factor here. It must be considered that the sampling point did not explain a large proportion of the variation in, and the dominance of, a single genus, *C. hepatoplasma*. Coupled with the lack of variation overall these results indicate that the hepatopancreas may have a stable microbiome that is not majorly influenced by external factors. Here using samples obtained from fisheries, both samples were fished in the same location however there may be slight variation in the precise placement of the fishing pots and we were unable to record environmental parameters during fishing activity; future studies would benefit from recording these parameters to help understand any differences observed.

Infection with the parasite *P. canceri* did not have a significant effect on variation in the microbiome. Several studies report the effects of parasite infection on crustacean microbiomes, such as in the shrimp *Penaeus vannamei* (López‐Carvallo et al., 2022), and *Litopenaeus setiferus* (Frischer et al., 2017). For both of these shrimp studies, it was noted that alterations in the microbiota structure or diversity occur during ‘peaks’ of infection, or more intense infections (Frischer et al., 2017; López‐Carvallo et al., 2022). Future studies could include a measure of infection intensity or parasite load to test for possible differences during low‐intensity infection compared to high‐intensity infections. The paramyxid *P. canceri*. can infect all tissues and organs and may contribute to host mortality but its impact on crab health is not yet well understood (Collins et al., 2022; Feist et al., 2009; Martin et al., 2024). However, if the hepatopancreas in particular has a more stable microbiome compared to other organs it may not be prone to changes in response to infection. There was also no variation in the microbiome explained by crab size or by the sex of the crab; previous examples of comparisons with size appear scarce, however previous studies have reported differences in microbiome composition between males and females in several species, including isopods *J. albifrons* (Wenzel et al., 2018), and mud crab *S. paramamosain* (Jiang et al., 2023).

The dominance of *C. hepatoplasma* within the top 25 most abundant ASVs is evidence of a low diversity microbiome, a finding further supported by the alpha diversity analyses where the average ASV evenness value was closer to zero than to one, indicating a low evenness community (Jost, 2010). In the intestinal microbiome of mud crab, *S. serrata*, evenness values were also closer to zero than to one for all samples (Apine et al., 2021). The average richness of the hepatopancreas microbiome of velvet crabs was 93 ASVs. García‐López et al. (2020) reported a lower richness in hepatopancreas microbiome compared to intestinal microbiome in cultured shrimp *L. vannamei*, while a study on signal crayfish *P. leniusculus* reported a similarly low richness from hepatopancreas, haemolymph, and intestine microbiomes compared to those from exoskeleton, water, and sediment (Dragičević et al., 2021). Direct comparisons of richness and other measures present a challenge due to variation in analyses between studies with differences in the use of either operational taxonomic units (OTUs) or ASVs, or studies using different measures of microbiome diversity, for instance, rarefied richness, as used in this study, versus using another method such as Shannon's diversity index which accounts for a combination of richness and abundance (Apine et al., 2021). On examining microbiome variation between samples, the beta diversity analyses showed clustering of samples collected at the two different sampling points, and a significant difference was found. These analyses demonstrate that within‐sample diversity was low, but that there was diversity between sampling groups, a pattern which follows results reported for the gut microbiome of the Chinese mitten crab, *E. sinensis* (Yu et al., 2021).

Examining the relationships between the microbial community and all study factors included here revealed the genera that were positively or negatively associated with each study factor. Focusing first on the top five ASVs most positively and negatively associated with sampling point, many of the ASVs were previously isolated from environmental samples such as water and/or were associated with pollution. Velvet crabs have an open circulatory system, which may assist in the transport of pollutants (Crooks et al., 2019; Yin et al., 2022). Caught in pot fisheries and fisheries capturing brown crab (Fahy et al., 2008; Hinchliff et al., 2015), these crabs have a targeted fishery in Galway Bay (Collins et al., 2022) which is a bay exposed to eutrophication, sewage effluent, and detritus (Allen, 2005). The bay experiences run‐off from several rivers (O'Brien, 1976), and includes run‐off from agricultural land and domestic waste (Yip, 1981). Future studies could investigate the extent to which these pollution sources may be associated with microbiota identified in the velvet crab hepatopancreas.

The ASVs most positively associated with Sampling point 1 included just one ASV, *Bdellovibrio*, that had previously been isolated from a crustacean, from both the haemolymph and hindgut of cultured juvenile spiny lobster *Panulirus ornatus* (Ooi et al., 2021), and has potential in crustacean aquaculture as a probiotic for general health (Liu et al., 2022) and as a treatment for vibriosis (Wen et al., 2009). Upon examining other covariates, there were also ASVs isolated from plant or algal sources that could be related to the opportunistic diet of velvet crab, including both animal material and algae, but predominantly consisting of brown algae (Norman, 1989; Norman & Jones, 1992). Diet has been reported to impact the microbiome, for instance, Zhang et al. (2020) reported diet as a factor shaping the gut microbiome of red swamp crayfish *Procambarus clarkii*. Two other ASVs associated with covariates had been associated with crustaceans before, and four were previously associated with disease or parasites (see Table S3 for more detail). Two ASVs in particular, *Halocynthiibacter* (positively associated with size) and *Thalassobius* (negatively associated with infection), were previously identified as important bacteria in shell diseases in decapod crustaceans (Bergen et al., 2022; Schaubeck et al., 2023).

A core microbiome was identified for the hepatopancreas of velvet crab in this study and the core phyla identified align with previous studies of the most abundant or core phyla in other crab microbiomes. The top four phyla (Pseudomonadota, Bacillota, Actinomycetota, Bacteroidota) appear as either the most abundant or core phyla in studies of the gut microbiome of Chinese mitten crab, *E. sinensis* (Yu et al., 2021) and in the gut and intestine microbiomes of the mud crabs *S. serrata* (Apine et al., 2021) and *S. paramamosain* (Jiang et al., 2023) Additionally, Actinomycetota, Bacteroidota, and Pseudomonadota appear as the core phyla on the carapace of brown crab, *C. pagurus* (Kraemer et al., 2020). The genera *Thiothrix* and *C. hepatoplasma* which were found to dominate the microbiota examined here were both identified as core ASVs, showing again the importance of these genera for the velvet crab hepatopancreas microbiome and their functions should be further investigated in these crabs. The selection processes behind the ASVs were investigated through the core analysis and *C. hepatoplasma* and *Thiothrix* were both identified as likely to be dispersal‐limited selected, which is a more passive process whereby individuals lost are replaced by organisms outside the local community (Burns et al., 2015). Those identified as likely to be host selected belonged to the four most abundant core phyla (Pseudomonadota, Bacillota, Actinomycetota, Bacteroidota), and additionally included Deinococcota. These ASVs range in their possible functions and the sources they have previously been isolated from. One of the classes to which ten of the ASVs belong is Actinobacteria which is implicated in maintaining gut homeostasis (Binda et al., 2018) and their presence could therefore benefit the velvet crab hepatopancreas. Also present was the order Micrococcales, suggested to protect against pathogens in mammals (Rojas‐Gätjens et al., 2022) and which could be of benefit to velvet crab also but requires further investigation.

The core microbiome was also identified across different occupancies. These results revealed distinctions between occupancies, in terms of the representation of some branches, i.e. differences in the number of unique taxa and counts or abundance of those taxa. In the occupancy Sampling point 2/infected/male, for example, there were several taxa better represented compared to other occupancies. Some of these taxa were previously associated with stony coral tissue loss disease (Rosales et al., 2020), the gut of the blue mussel, *Mytilus edulis* (Li et al., 2020) and white shrimp *P. vannamei* (Amin et al., 2024), and the crab *Atergatis reticulatus* (Yang et al., 2017). The distinctions between occupancies may be associated with a range of factors, with differences in crustacean microbiome composition previously recorded between males and females (Wenzel et al., 2018), with different time points (Yu et al., 2021), and with parasite infection (Frischer et al., 2017). Compositional differences have also been reported to vary with temperature (Apine et al., 2021), salinity (Saqib et al., 2023), and host moult stage (Mente et al., 2016; Zhang et al., 2021), which can all vary with time and therefore the two different timepoints in this study could represent a number of factors within them that change and impact the microbiome composition. Further investigations are needed to shed light on the explanations for these associations.

We established microbiome taxa composition and analysed diversity for the hepatopancreas of the velvet crab, *N. puber*. We found that the hepatopancreas had a low diversity of microbiota overall and was dominated by *C. hepatoplasma*, a possible gut symbiont. We identified the presence of a core microbiome which allowed further investigations into the implications and sources of various ASVs as reported in the literature. The hepatopancreas microbiome here appears stable and without a lot of variation, but further investigation is needed on the factors affecting the microbiome of velvet crabs. In particular, investigations into the effects of parasite infection on the microbial composition and structure should aim for more even sample sizes of infected versus uninfected hosts and could include a measure of infection intensity. Here, the sampling point was identified as the only source of variation of the study factors, but it explained only a small percentage of the variation and the inclusion of more time points and locations, along with the inclusion of environmental data, would be beneficial in future. In addition, performing a quantitative PCR to obtain absolute abundances would give further clarification and robustness in future investigations. The research carried out provides a first description of the microbiome of velvet crab hepatopancreas, providing a baseline for future investigations of factors affecting the microbial composition and effects on the health of velvet crab.

## AUTHOR CONTRIBUTIONS

**Signe Martin:** Conceptualization; methodology; data curation; investigation; formal analysis; validation; visualization; project administration; writing – original draft; writing – review and editing; software. **Cindy Smith:** Conceptualization; methodology; data curation; supervision; resources; formal analysis; project administration; validation; writing – review and editing. **Kelly Stewart:** Investigation; formal analysis; methodology. **Deborah Cheslett:** Conceptualization; methodology; project administration; resources; supervision; writing – review and editing. **Ian O'Connor:** Conceptualization; funding acquisition; methodology; project administration; resources; supervision; writing – review and editing. **Fiona Swords:** Conceptualization; investigation; methodology; project administration; resources; supervision; writing – review and editing. **Umer Zeeshan Ijaz:** Data curation; formal analysis; conceptualization; methodology; resources; software; validation; visualization; writing – original draft; writing – review and editing. **Katie O'Dwyer:** Conceptualization; data curation; funding acquisition; supervision; project administration; methodology; resources; validation; writing – review and editing; writing – original draft. **William Barr:** Formal analysis; visualization; methodology; software; writing – review and editing.

## CONFLICT OF INTEREST STATEMENT

The authors declare no conflicts of interest.

## ETHICS STATEMENT

Formal ethical approval was not required, and the study followed recommendations for good animal welfare.

## Supporting information


**Data S1.** Supporting information.

**Figure S2.** (i) β‐Coefficients returned for individual genera from the GLLVM procedure against the sources of variation considered in this study (size, infection status, sampling point, sex). The results continue to Figure S2 (ii–ix).

**Figure S4.** (i) Taxonomic coverage tree of the core velvet crab hepatopancreas microbiome showing collated abundances across all occupancies (sampling point [1/2], infection with *Paramarteilia canceri* [Yes/No], and sex [Male/Female]). The key on the right side of the tree can be interpreted as follows: the width of the bar represents the number of unique taxa and is the size of the nodes (shown on the left side of the key), while the colour represents the count of these taxa (shown on the right side of the key). (ii) Taxonomic coverage tree of the core velvet crab hepatopancreas microbiome for the occupancy; Sampling point 1; Not infected; Female. Interpretation is the same as what is mentioned in the legend of Figure S4, i. (iii) Taxonomic coverage tree of the core velvet crab hepatopancreas microbiome for the occupancy; Sampling point 1; Not infected; Male. Interpretation is the same as what is mentioned in the legend of Figure S4, i. (iv) Taxonomic coverage tree of the core velvet crab hepatopancreas microbiome for the occupancy; Sampling point 1; Infected; Female. Interpretation is the same as what is mentioned in the legend of Figure S4, i. (v) Taxonomic coverage tree of the core velvet crab hepatopancreas microbiome for the occupancy; Sampling point 1; Infected; Male. Interpretation is the same as what is mentioned in the legend of Figure S4, i. (vi) Taxonomic coverage tree of the core velvet crab hepatopancreas microbiome for the occupancy; Sampling point 2; Not infected; Female. Interpretation is the same as what is mentioned in the legend of Figure S4, i. (vii) Taxonomic coverage tree of the core velvet crab hepatopancreas microbiome for the occupancy; Sampling point 2; Infected; Female. Interpretation is the same as what is mentioned in the legend of Figure S4, i. (viii) Taxonomic coverage tree of the core velvet crab hepatopancreas microbiome for the occupancy; Sampling point 2; Infected; Male. Interpretation is the same as what is mentioned in the legend of Figure S4, i.

Table S4.

## Data Availability

The raw sequence files supporting the results of this article are available in the European Nucleotide Archive under the project accession number PRJEB72630 with details of the samples provided in Supplementary_Data_Table_S4.csv (Table S4). Some of the code used to generate the results are part of microbiomeSeq (https://github.com/umerijaz/microbiomeSeq), while other codes are available at http://userweb.eng.gla.ac.uk/umer.ijaz#bioinformatics. Supplementary_Data_Table_S4.csv is the metadata table which provides the details of the samples on which the raw sequence files are based (these raw sequence files supporting the results of this article are available in the European Nucleotide Archive under the project accession number PRJEB72630). The data can be interpreted as follows: Sample_ID: F = forward primer number, R = reverse primer number (see Table S1 for more detail on primer combinations), 14 or 15 refers to the month number the samples were collected as part of another study (Martin et al., 2024), G = Galway Bay, V = velvet crab, the following number refers to the crab number sampled in a single month's sample (1–30). Sample_or_Control refers to whether it was a true sample or a negative control sample. The control samples are either ‘NPC’, a non‐processed blank DNA extraction control, or NC refers to blank PCR controls. Infected: Y = infected with *Paramarteilia canceri*, N = not infected with *P. canceri*. The sampling date is the day crabs were collected from the fisher that provided the samples, season refers to this sampling date also. Sex: M = male crab, F = female crab. CW = carapace width of the crabs in millimetres. Quant_reading = Nanodrop quantification (ng/μL) of the purified PCR products before sending for sequencing.
