# Supplementary material for: The hepatopancreas microbiome of velvet crab, Necora puber
Source: Environ Microbiol Rep. 2024 Oct 1;16(5):e70014. doi: 10.1111/1758-2229.70014 (PMC11445078; doi:10.1111/1758-2229.70014)
Supplement: Supplementary file 1 — Data S1. Supporting information. [file EMI4-16-e70014-s004.docx]

APPENDIX

Table S1. Primer combinations used to amplify the V1/V2 region of the 16S rRNA gene. The exact sequences of the combination of forward and reverse primers are displayed along with the number of samples the primer combination was used to amplify. NPC refers to non-processed blank DNA extraction controls, while NC refers to blank PCR controls.

| **Primer combination** | **No. samples** | **No. controls** | **F primer sequence (barcode + index + adaptor sequence + primer sequence)** | **R primer sequence (adaptor sequence + index + spacer + primer sequence)** |
| --- | --- | --- | --- | --- |
| F1R3 | 3 | 1 NPC | ACACTCTTTCCCTACACGACGCTCTTCCGATCT + CCTAAACTACGG + AGAGTTTGATCM TGGCTCAG | GACTGGAGTTCAGACGTGTGCTCTTCCGATCT + CCATCACATAGG + GCTGCCTCCCGTAGGAGT |
| F2R2 | 4 |  | ACACTCTTTCCCTACACGACGCTCTTCCGATCT + TGCAGATCCAAC + AGAGTTTGATCM TGGCTCAG | GACTGGAGTTCAGACGTGTGCTCTTCCGATCT + TGCAGATCCAAC + GCTGCCTCCCGTAGGAGT |
| F2R3 | 4 | 1 NPC | ACACTCTTTCCCTACACGACGCTCTTCCGATCT + TGCAGATCCAAC + AGAGTTTGATCM TGGCTCAG | GACTGGAGTTCAGACGTGTGCTCTTCCGATCT + CCATCACATAGG + GCTGCCTCCCGTAGGAGT |
| F3R4 | 5 | 1 NPC, 1 NC | ACACTCTTTCCCTACACGACGCTCTTCCGATCT + CCATCACATAGG + AGAGTTTGATCM TGGCTCAG | GACTGGAGTTCAGACGTGTGCTCTTCCG ATCT + GTGGTATGGGAG + **A** + GCTGCCTCCCGTAGGAGT |
| F3R5 | 4 | 1 NC | ACACTCTTTCCCTACACGACGCTCTTCCGATCT + CCATCACATAGG + AGAGTTTGATCM TGGCTCAG | GACTGGAGTTCAGACGTGTGCTCTTCCG ATCT + ACTTTAAGGGTG + **A** + GCTGCCTCCCGTAGGAGT |
| F3R6 | 10 | 1 NPC | ACACTCTTTCCCTACACGACGCTCTTCCGATCT + CCATCACATAGG + AGAGTTTGATCM TGGCTCAG | GACTGGAGTTCAGACGTGTGCTCTTCCG ATCT + GAGCAACATCCT + **A** + GCTGCCTCCCGTAGGAGT |

Text to support Table S2.

The table contains the PERMANOVA with RDA results using Unweighted UniFrac distance examining potential factors driving variation in the microbial community. The sampling point was identified as the only significant factor driving variation.

Table S2. PERMANOVA with RDA results using Unweighted UniFrac distance. Significant values are indicated in bold.

|  | **Covariates** | **Degrees of freedom** | **Sum of Squares** | **R^2^** | **F** | **p-value** |
| --- | --- | --- | --- | --- | --- | --- |
| Unweighted  UniFrac Distance | Sampling point | 1 | 0.534 | 0.062 | 1.717 | **0.005** |
|  | Infection | 1 | 0.346 | 0.040 | 1.111 | 0.169 |
|  | Crab sex | 1 | 0.297 | 0.035 | 0.955 | 0.594 |
|  | Crab size | 1 | 0.271 | 0.032 | 0.872 | 0.775 |
|  | Residual | 23 | 7.159 | 0.832 |  |  |
|  | Total | 27 | 8.607 | 1.000 |  |  |

Text to support Figure S1:

Beta diversity demonstrated a clustering of the two different sampling points, and a significant difference was found between them in the Unweighted UniFrac distance (R^2^ = 0.06, p = 0.006, Fig. S1).

Figure S1. Beta diversity index showing Bray-Curtis dissimilarity matrix results with PERMANOVA R2 (Percentage variability) and p values provided below the plot.

Text to support Figure S2:

The results of the Generalised Linear Latent Variable Model (GLLVM) revealed which microbes are positively and negatively associated with each of the study factors. ASVs positively associated with size were more abundant in larger crabs, those associated with infected: yes, were more abundant in infected crabs, those associated with sampling point one were more abundant in samples from sampling point one, and those positively associated with Sex: female was more abundant in female crabs. Positively associated ASVs are indicated by a blue “x”, and negatively associated ASVs with a red “x”. Those microbes which were statistically insignificant, i.e., where the coefficients crossed the 0 boundary, are greyed out. All those ASVs that could not be categorised based on taxonomy at the genus level are collated under the “__Unknowns__” category.

Figure S2 i. β-coefficients returned for individual genera from the GLLVM procedure against the sources of variation considered in this study (size, infection status, sampling point, sex). The results continue to Figures S2 ii - ix.

Figure S2 ii. Continuation of results from Figure S2 for the β-coefficients returned from the GLLVM procedure.

Figure S2 iii. Continuation of results from Figure S2 for the β-coefficients returned from the GLLVM procedure.

Figure S2 iv. Continuation of results from Figure S2 for the β-coefficients returned from the GLLVM procedure.

Figure S2 v. Continuation of results from Figure S2 for the β-coefficients returned from the GLLVM procedure.

Figure S2 vi. Continuation of results from Figure S2 for the β-coefficients returned from the GLLVM procedure.

Figure S2 vii. Continuation of results from Figure S2 for the β-coefficients returned from the GLLVM procedure.

Figure S2 viii. Continuation of results from Figure S2 for the β-coefficients returned from the GLLVM procedure.

Figure S2 ix. Continuation of results from Figure S2 for the β-coefficients returned from the GLLVM procedure.

Text to support Table S3:

The results of the Generalised Linear Latent Variable Model (GLLVM) revealed which microbes are positively and negatively associated with each of the study factors. We further investigated in the literature the top five most positively and negatively associated microbes with each covariate, identifying previous isolation sources.

Table S3. The top five most positively and negatively associated microbes with each covariate and previous isolation sources identified by literature screening.

| **ASV** | **Positively associated covariates** | **Negatively associated covariates** | **Isolation source** | **Reference** |
| --- | --- | --- | --- | --- |
| Lentilitoribacter | Sampling point | Size, Sex, Infection | animal (fish) | (Park et al. 2013, Méndez-Pérez et al. 2019) |
| Candidatus Moranbacteria | Size, Sex, Sampling point |  | environment (water) | (Wagner et al. 2009, Han et al. 2010) |
| Undibacterium | Size, Sex |  | environment (water), animal (mammal, insect) | (Kim et al. 2014, Zhang et al. 2022, Schaubeck et al. 2023) |
| Gracilibacteria |  | Size, Sex | environment (water) | (Figueroa-Gonzalez et al. 2023) |
| Tepidimonas |  | Size, Sampling point | animal (fish),  environment (water) | (Chen et al. 2013, Wang et al. 2020a) |
| Effusibacillus | Infection | Sampling point | environment (sediment) | (Konishi et al. 2021) |
| Schlegelella | Size |  | unclear |  |
| Caldanaerobius | Size |  | environment (water) |  |
| Halocynthiibacter | Size |  | animal (crustacean), animal (invertebrates) | (Kim et al. 2014, Zhang et al. 2022, Schaubeck et al. 2023) |
| Sulfurovum |  | Size | unclear |  |
| Robiginitomaculum |  | Size | algae | (Ramírez-Puebla et al. 2022) |
| Parcubacteria | Infection |  | environment (anoxic) | (Nelson & Stegen 2015) |
| Rhodobacter | Infection |  | environment (water, sediment) | (Subhash & Lee 2016, Chen et al. 2021) |
| WPS−2 | Infection |  | environment (soil) plants | (Holland‐Moritz et al. 2018, Sheremet et al. 2020) |
| Candidatus Kerfeldbacteria | Infection |  | environment (water, biofilms) | (Casar et al. 2021, Ettinger et al. 2021) |
| Ruegeria |  | Infected | unclear | (Arahal et al. 2018) |
| Rhizobium |  | Infected | plant, algae | (Johnston & Behringer 1975, Wasai & Minamisawa 2018, Miller et al. 2022) |
| Thalassobius |  | Infected | animal (crustacean)  + disease | (Chong 2022) |
| Parvibaculales |  | Infected | animal (sponge) | (Baquiran et al. 2020) |
| Cellvibrio | Sex |  | environment (soil) | (Sato et al. 2010) |
| Acidimicrobiia | Sex |  | animal (crustacean) | (Bergen et al. 2022) |
| Cytophaga | Sex |  | animal (fish) | (Wakabayashi et al. 1994) |
| Bdellovibrionaceae, OM27_clade |  | Sex | unclear |  |
| Comamonas |  | Sex | animal (invertebrates), environment (soil) | (Hatayama 2014, Coon et al. 2020) |
| Photobacterium |  | Sex | animal (crustacean)  + disease | (Wang et al. 2020b, Xie et al. 2021) |
| Flavobacteriales, NS9_marine_group | Season |  | unclear | (Yeh & Fuhrman 2022) |
| Brevibacillus | Season |  | unclear | (Wang et al. 2021) |
| Iamia | Season |  | animal (invertebrate) | (Kurahashi et al. 2009) |
| Janibacter |  | Season | environment | (Elsayed & Zhang 2005) |
| Xylella |  | Season | plant | (Saddler & Bradbury 2015, Sicard et al. 2018) |
| Bdellovibrio |  | Season | environment (water), animal (crustacean) | (Wen et al. 2009) |

Text to support Figure S3

A core microbiome was identified in the velvet crab hepatopancreas of 12 phyla, and their proportions of the core microbiome can be seen in Figure S3. A neutral model was fitted to the “S” shaped abundance-occupancy distributions and those that fall outside the 95% confidence interval of the fitted model are those considered not neutrally selected, those that are above the model are indicated as selected by the host environment, and those that are below the model indicated as selected by dispersal limitation. For each core phyla, we identify whether they are neutral, likely to be selected by the host environment (above), or deterministically assembled (below) (Fig. S4).


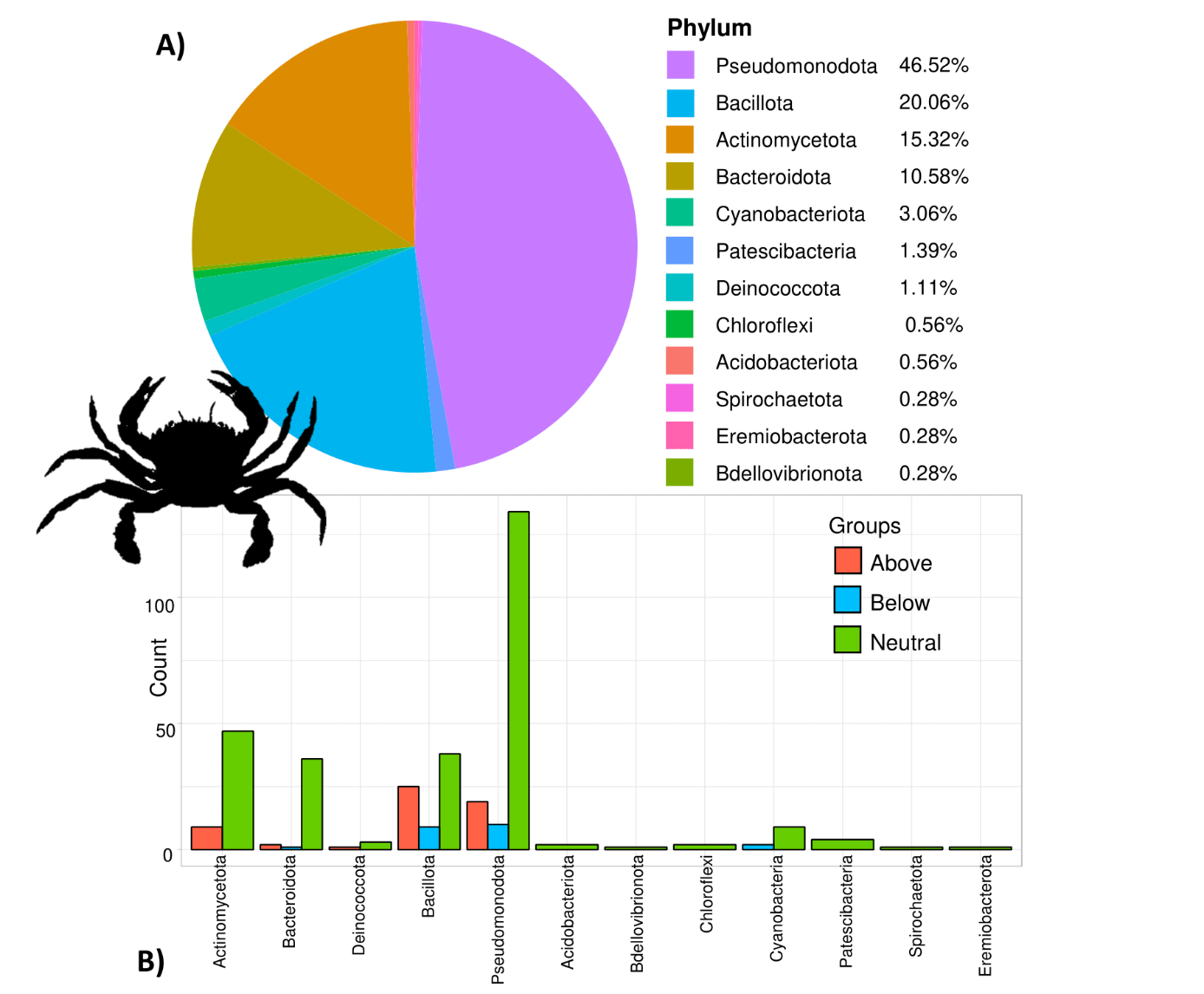


Figure S3. A) Velvet crab hepatopancreas microbiome core phyla. B) Velvet crab hepatopancreas core phyla showing whether they are above (host selected), neutral, or below (dispersal limited) the predictions by the neutral model.

Text to support Figure S4

The core microbiome calculation used an occupancy model, allowing for investigation of the velvet crab hepatopancreas core ASVs at different site-specific occupancies using taxonomic coverage trees. These trees of the core microbiome show collated abundances across i) all occupancies (sampling point [1/2], infection with *Paramarteilia canceri* [Yes/No], and sex [Male/Female] collated), and ii – viii) the seven different occupancies: SP:1 I:N S:F; SP:1 I:N S:M; SP:1 I:Y S:F; SP:1 I:Y S:M; SP:2 I:N S:F; SP:2 I:Y S:F; SP:2 I:Y S:M.

Figure S4 i). Taxonomic coverage tree of the core velvet crab hepatopancreas microbiome showing collated abundances across all occupancies (sampling point [1/2], infection with *Paramarteilia canceri* [Yes/No], and sex [Male/Female]). The key on the right side of the tree can be interpreted as follows: the width of the bar represents the number of unique taxa and is the size of the nodes (shown on the left side of the key), whilst the colour represents the count of these taxa (shown on the right side of the key).

Figure S4 ii). Taxonomic coverage tree of the core velvet crab hepatopancreas microbiome for the occupancy; Sampling point 1; Not infected; Female. Interpretation is the same as what is mentioned in the legend of Figure S4 i).

Figure S4 iii). Taxonomic coverage tree of the core velvet crab hepatopancreas microbiome for the occupancy; Sampling point 1; Not infected; Male. Interpretation is the same as what is mentioned in the legend of Figure S4 i).

Figure S4 iv). Taxonomic coverage tree of the core velvet crab hepatopancreas microbiome for the occupancy; Sampling point 1; Infected; Female. Interpretation is the same as what is mentioned in the legend of Figure S4 i).

Figure S4 v) Taxonomic coverage tree of the core velvet crab hepatopancreas microbiome for the occupancy; Sampling point 1; Infected; Male. Interpretation is the same as what is mentioned in the legend of Figure S4 i).

Figure S4 vi). Taxonomic coverage tree of the core velvet crab hepatopancreas microbiome for the occupancy; Sampling point 2; Not infected; Female. Interpretation is the same as what is mentioned in the legend of Figure S4 i).

Figure S4 vii). Taxonomic coverage tree of the core velvet crab hepatopancreas microbiome for the occupancy; Sampling point 2; Infected; Female. Interpretation is the same as what is mentioned in the legend of Figure S4 i).

Figure S4 viii). Taxonomic coverage tree of the core velvet crab hepatopancreas microbiome for the occupancy; Sampling point 2; Infected; Male. Interpretation is the same as what is mentioned in the legend of Figure S4 i).
