# Supplementary material for: The hepatopancreas microbiome of velvet crab, Necora puber
Source: Environ Microbiol Rep. 2024 Oct 1;16(5):e70014. doi: 10.1111/1758-2229.70014 (PMC11445078; doi:10.1111/1758-2229.70014)
Supplement: Supplementary file 2 — Figure S2. (i) β‐Coefficients returned for individual genera from the GLLVM procedure against the sources of variation considered in this study (size, infection status, sampling point, sex). The results continue to Figure S2 (ii–ix). [file EMI4-16-e70014-s003.pdf]

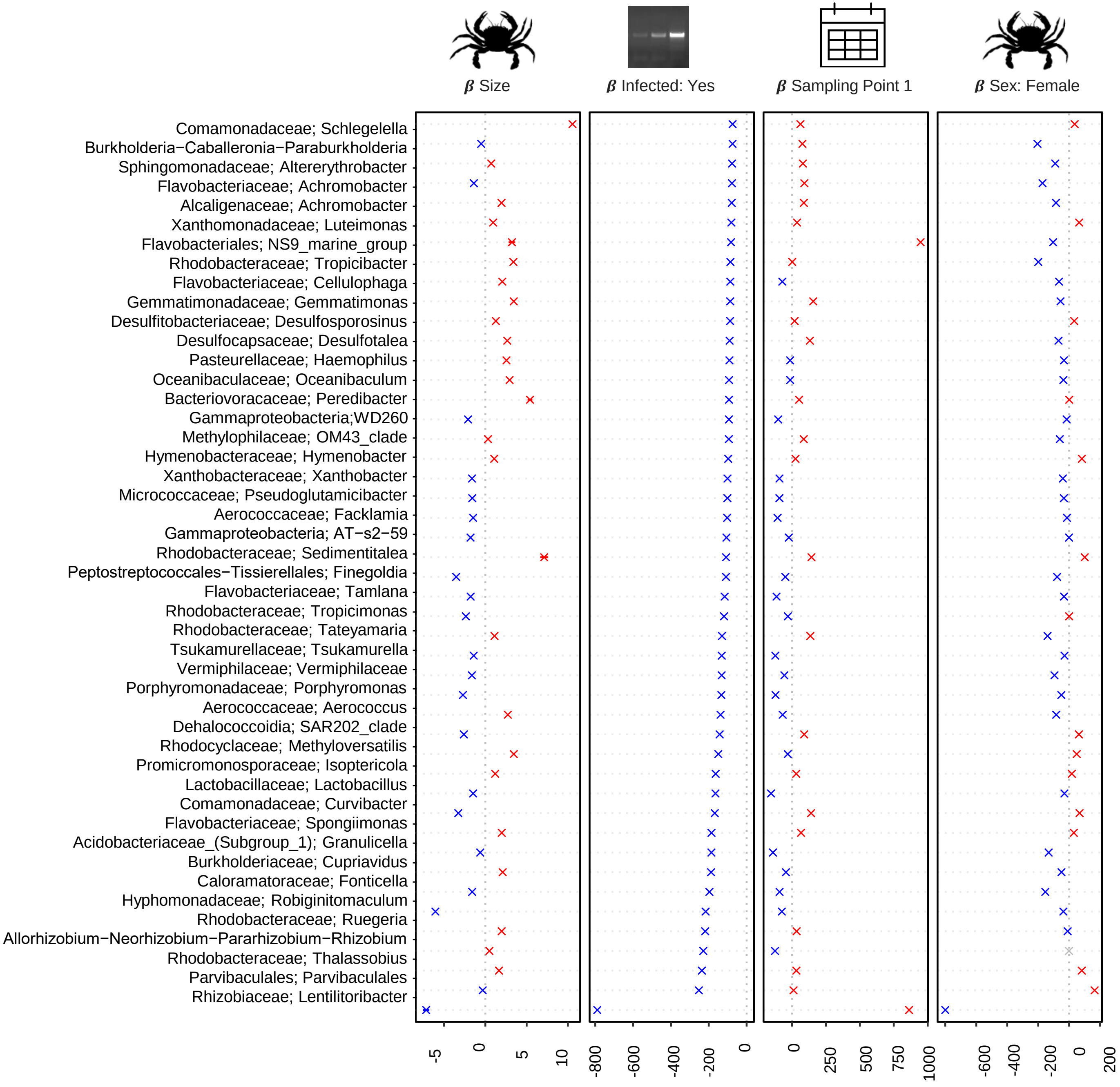

β Size
β Infected: Yes
β Sampling Point 1
β Sex: Female
Comamonadaceae; Schlegelella
Burkholderia−Caballeronia−Paraburkholderia
Sphingomonadaceae; Altererythrobacter
Flavobacteriaceae; Achromobacter
Alcaligenaceae; Achromobacter
Xanthomonadaceae; Luteimonas
Flavobacteriales; NS9_marine_group
Rhodobacteraceae; Tropicibacter
Flavobacteriaceae; Cellulophaga
Gemmatimonadaceae; Gemmatimonas
Desulfitobacteriaceae; Desulfosporosinus
Desulfocapsaceae; Desulfotalea
Pasteurellaceae; Haemophilus
Oceanibaculaceae; Oceanibaculum
Bacteriovoracaceae; Peredibacter
Gammaproteobacteria;WD260
Methylophilaceae; OM43_clade
Hymenobacteraceae; Hymenobacter
Xanthobacteraceae; Xanthobacter
Micrococcaceae; Pseudoglutamicibacter
Aerococcaceae; Facklamia
Gammaproteobacteria; AT−s2−59
Rhodobacteraceae; Sedimentitalea
Peptostreptococcales−Tissierellales; Finegoldia
Flavobacteriaceae; Tamlana
Rhodobacteraceae; Tropicimonas
Rhodobacteraceae; Tateyamaria
Tsukamurellaceae; Tsukamurella
Vermiphilaceae; Vermiphilaceae
Porphyromonadaceae; Porphyromonas
Aerococcaceae; Aerococcus
Dehalococcoidia; SAR202_clade
Rhodocyclaceae; Methyloversatilis
Promicromonosporaceae; Isoptericola
Lactobacillaceae; Lactobacillus
Comamonadaceae; Curvibacter
Flavobacteriaceae; Spongiimonas
Acidobacteriaceae_(Subgroup_1); Granulicella
Burkholderiaceae; Cupriavidus
Caloramatoraceae; Fonticella
Hyphomonadaceae; Robiginitomaculum
Rhodobacteraceae; Ruegeria
Allorhizobium−Neorhizobium−Pararhizobium−Rhizobium
Rhodobacteraceae; Thalassobius
Parvibaculales; Parvibaculales
Rhizobiaceae; Lentilitoribacter
-5
0
5
10
-800
-600
-400
-200
0
0
250
500
750
1000
-600
-400
-200
0
200

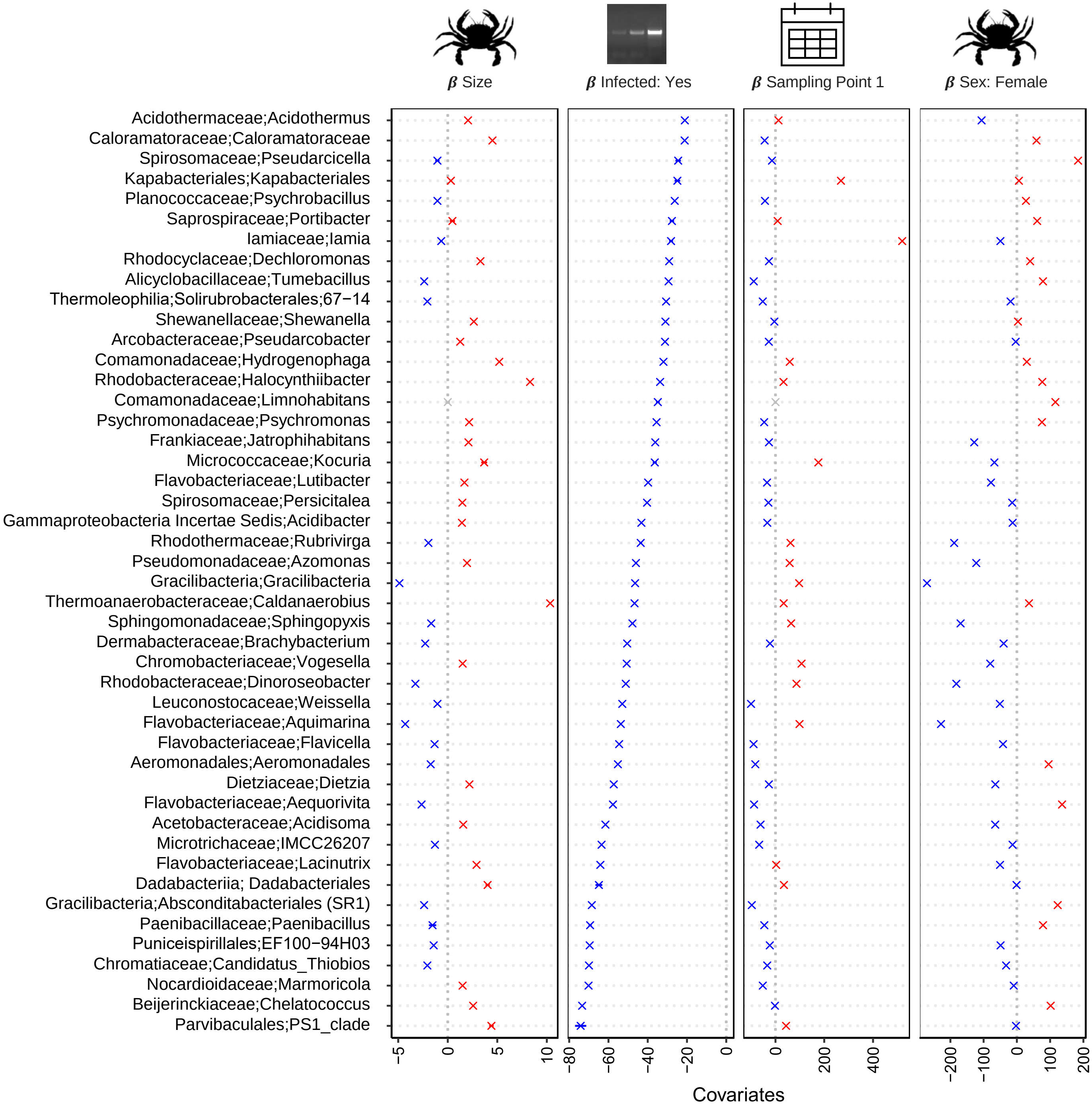
β Size
β Infected: Yes
β Sampling Point 1
β Sex: Female
Acidothermaceae;Acidothermus
Caloramatoraceae;Caloramatoraceae
Spirosomaceae;Pseudarcicella
Kapabacteriales;Kapabacteriales
Planococcaceae;Psychrobacillus
Saprospiraceae;Portibacter
Iamiaceae;Iamia
Rhodocyclaceae;Dechloromonas
Alicyclobacillaceae;Tumebacillus
Thermoleophilia;Solirubrobacterales;67−14
Shewanellaceae;Shewanella
Arcobacteraceae;Pseudarcobacter
Comamonadaceae;Hydrogenophaga
Rhodobacteraceae;Halocynthiibacter
Comamonadaceae;Limnohabitans
Psychromonadaceae;Psychromonas
Frankiaceae;Jatrophihabitans
Micrococcaceae;Kocuria
Flavobacteriaceae;Lutibacter
Spirosomaceae;Persicitalea
Gammaproteobacteria Incertae Sedis;Acidibacter
Rhodothermaceae;Rubrivirga
Pseudomonadaceae;Azomonas
Gracilibacteria;Gracilibacteria
Thermoanaerobacteraceae;Caldanaerobius
Sphingomonadaceae;Sphingopyxis
Dermabacteraceae;Brachybacterium
Chromobacteriaceae;Vogesella
Rhodobacteraceae;Dinoroseobacter
Leuconostocaceae;Weissella
Flavobacteriaceae;Aquimarina
Flavobacteriaceae;Flavicella
Aeromonadales;Aeromonadales
Dietziaceae;Dietzia
Flavobacteriaceae;Aequorivita
Acetobacteraceae;Acidisoma
Microtrichaceae;IMCC26207
Flavobacteriaceae;Lacinutrix
Dadabacteriia; Dadabacteriales
Gracilibacteria;Absconditabacteriales (SR1)
Paenibacillaceae;Paenibacillus
Puniceispirillales;EF100−94H03
Chromatiaceae;Candidatus_Thiobios
Nocardioidaceae;Marmoricola
Beijerinckiaceae;Chelatococcus
Parvibaculales;PS1_clade
−5
0
5
10
−80
−60
−40
−20
0
0
200
400
−200
−100
0
100
200
Covariates

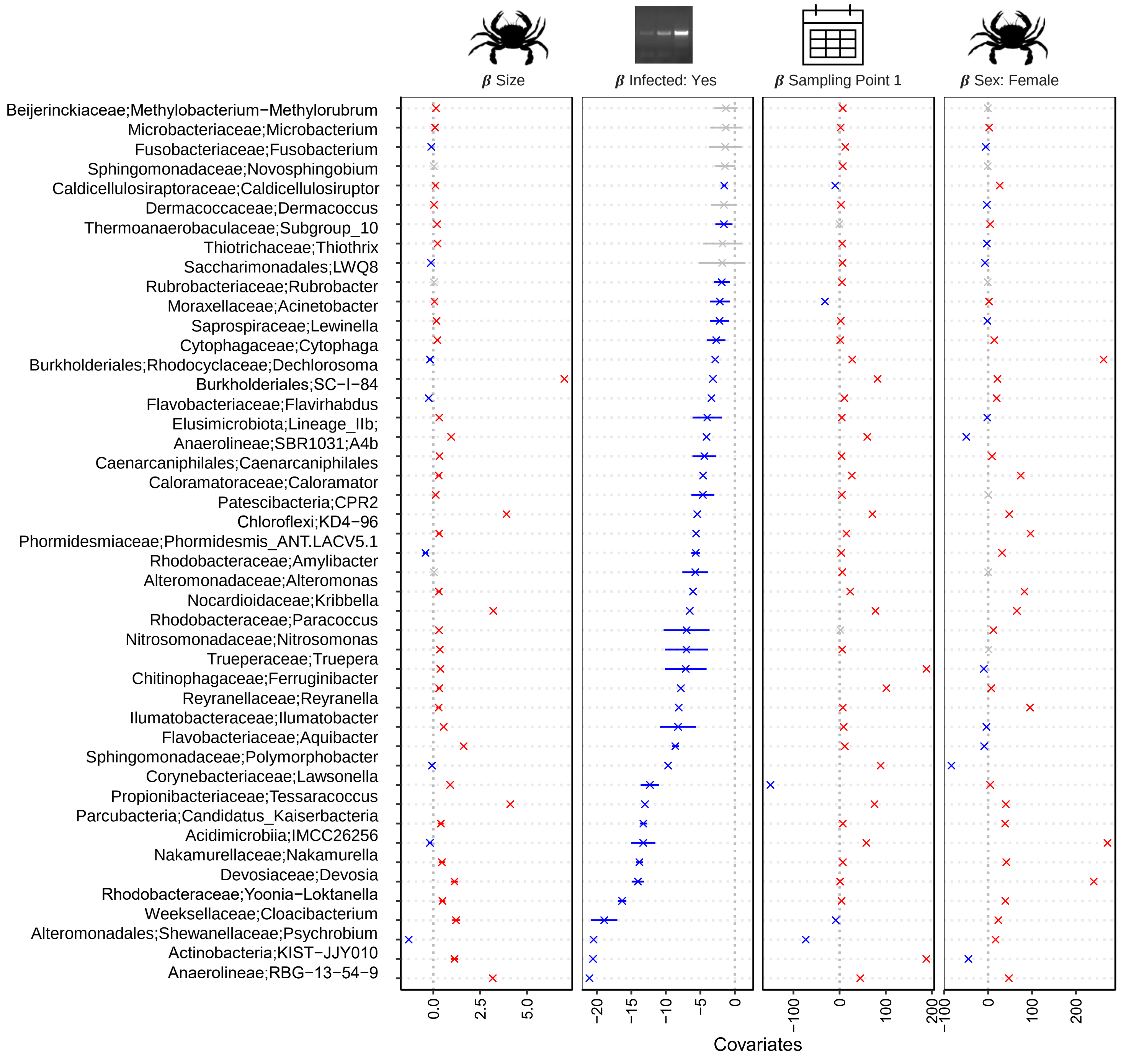

β Size
β Infected: Yes
β Sampling Point 1
β Sex: Female
Beijerinckiaceae;Methylobacterium−Methylorubrum
Microbacteriaceae;Microbacterium
Fusobacteriaceae;Fusobacterium
Sphingomonadaceae;Novosphingobium
Caldicellulosiraptoraceae;Caldicellulosiruptor
Dermacoccaceae;Dermacoccus
Thermoanaerobaculaceae;Subgroup_10
Thiotrichaceae;Thiothrix
Saccharimonadales;LWQ8
Rubrobacteriaceae;Rubrobacter
Moraxellaceae;Acinetobacter
Saprospiraceae;Lewinella
Cytophagaceae;Cytophaga
Burkholderiales;Rhodocyclaceae;Dechlorosoma
Burkholderiales;SC−I−84
Flavobacteriaceae;Flavirhabdus
Elusimicrobiota;Lineage_IIb;
Anaerolineae;SBR1031;A4b
Caenarcaniphilales;Caenarcaniphilales
Caloramatoraceae;Caloramator
Patescibacteria;CPR2
Chloroflexi;KD4−96
Phormidesmiaceae;Phormidesmis_ANT.LACV5.1
Rhodobacteraceae;Amylibacter
Alteromonadaceae;Alteromonas
Nocardioidaceae;Kribbella
Rhodobacteraceae;Paracoccus
Nitrosomonadaceae;Nitrosomonas
Trueperaceae;Truepera
Chitinophagaceae;Ferruginibacter
Reyranellaceae;Reyranella
Ilumatobacteraceae;Ilumatobacter
Flavobacteriaceae;Aquibacter
Sphingomonadaceae;Polymorphobacter
Corynebacteriaceae;Lawsonella
Propionibacteriaceae;Tessaracoccus
Parcubacteria;Candidatus_Kaiserbacteria
Acidimicrobiia;IMCC26256
Nakamurellaceae;Nakamurella
Devosiaceae;Devosia
Rhodobacteraceae;Yoonia−Loktanella
Weeksellaceae;Cloacibacterium
Alteromonadales;Shewanellaceae;Psychrobium
Actinobacteria;KIST−JJY010
Anaerolineae;RBG−13−54−9
0.0
2.5
5.0
−20
−15
−10
−5
0
−100
0
100
200
−100
0
100
200
Covariates

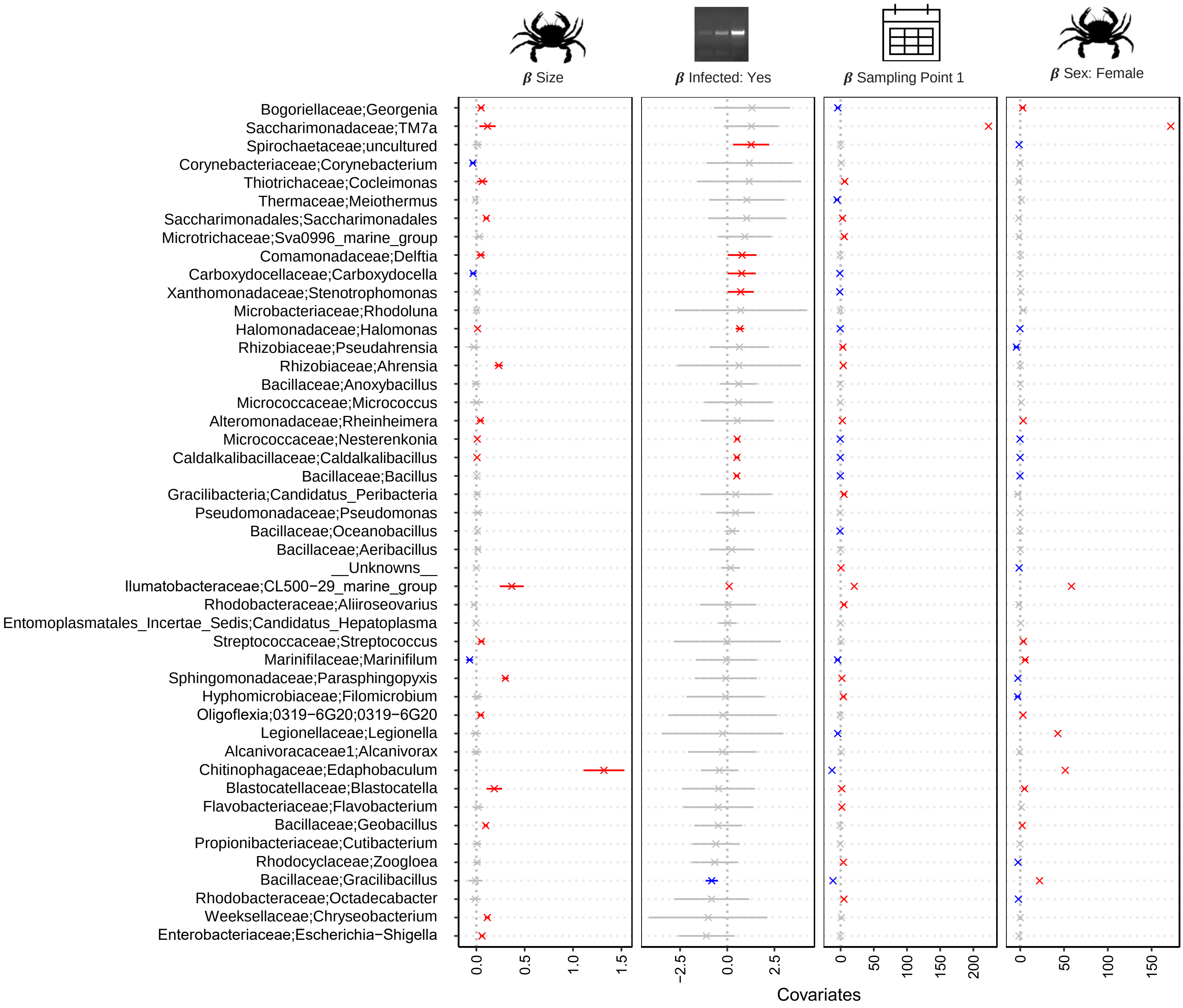

β Size
β Infected: Yes
β Sampling Point 1
β Sex: Female
Bogoriellaceae;Georgenia
Saccharimonadaceae;TM7a
Spirochaetaceae;uncultured
Corynebacteriaceae;Corynebacterium
Thiotrichaceae;Cocleimonas
Thermaceae;Meiothermus
Saccharimonadales;Saccharimonadales
Microtrichaceae;Sva0996_marine_group
Comamonadaceae;Delftia
Carboxydocellaceae;Carboxydocella
Xanthomonadaceae;Stenotrophomonas
Microbacteriaceae;Rhodoluna
Halomonadaceae;Halomonas
Rhizobiaceae;Pseudahrensia
Rhizobiaceae;Ahrensia
Bacillaceae;Anoxybacillus
Micrococcaceae;Micrococcus
Alteromonadaceae;Rheinheimera
Micrococcaceae;Nesterenkonia
Caldalkalibacillaceae;Caldalkalibacillus
Bacillaceae;Bacillus
Gracilibacteria;Candidatus_Peribacteria
Pseudomonadaceae;Pseudomonas
Bacillaceae;Oceanobacillus
Bacillaceae;Aeribacillus
__Unknowns__
Ilumatobacteraceae;CL500−29_marine_group
Rhodobacteraceae;Aliiroseovarius
Entomoplasmatales_Incertae_Sedis;Candidatus_Hepatoplasma
Streptococcaceae;Streptococcus
Marinifilaceae;Marinifilum
Sphingomonadaceae;Parasphingopyxis
Hyphomicrobiaceae;Filomicrobium
Oligoflexia;0319−6G20;0319−6G20
Legionellaceae;Legionella
Alcanivoracaceae1;Alcanivorax
Chitinophagaceae;Edaphobaculum
Blastocatellaceae;Blastocatella
Flavobacteriaceae;Flavobacterium
Bacillaceae;Geobacillus
Propionibacteriaceae;Cutibacterium
Rhodocyclaceae;Zoogloea
Bacillaceae;Gracilibacillus
Rhodobacteraceae;Octadecabacter
Weeksellaceae;Chryseobacterium
Enterobacteriaceae;Escherichia−Shigella
0.0
0.5
1.0
1.5
−2.5
0.0
2.5
0
50
100
150
200
0
50
100
150
Covariates

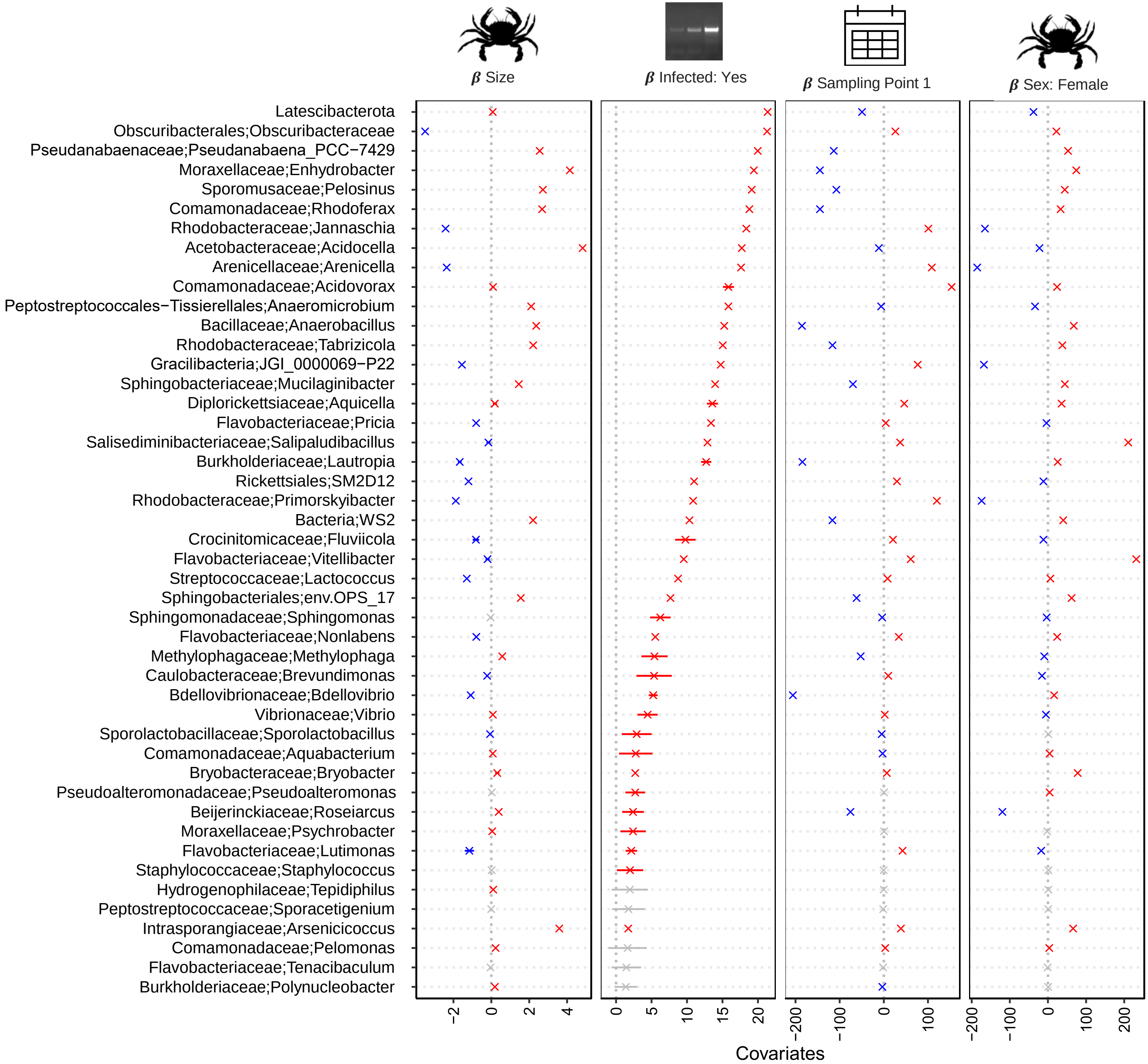

β Size
β Infected: Yes
β Sampling Point 1
β Sex: Female
Latescibacterota
Obscuribacterales;Obscuribacteraceae
Pseudanabaenaceae;Pseudanabaena_PCC−7429
Moraxellaceae;Enhydrobacter
Sporomusaceae;Pelosinus
Comamonadaceae;Rhodoferax
Rhodobacteraceae;Jannaschia
Acetobacteraceae;Acidocella
Arenicellaceae;Arenicella
Comamonadaceae;Acidovorax
Peptostreptococcales−Tissierellales;Anaeromicrobium
Bacillaceae;Anaerobacillus
Rhodobacteraceae;Tabrizicola
Gracilibacteria;JGI_0000069−P22
Sphingobacteriaceae;Mucilaginibacter
Diplorickettsiaceae;Aquicella
Flavobacteriaceae;Pricia
Salisediminibacteriaceae;Salipaludibacillus
Burkholderiaceae;Lautropia
Rickettsiales;SM2D12
Rhodobacteraceae;Primorskyibacter
Bacteria;WS2
Crocinitomicaceae;Fluviicola
Flavobacteriaceae;Vitellibacter
Streptococcaceae;Lactococcus
Sphingobacteriales;env.OPS_17
Sphingomonadaceae;Sphingomonas
Flavobacteriaceae;Nonlabens
Methylophagaceae;Methylophaga
Caulobacteraceae;Brevundimonas
Bdellovibrionaceae;Bdellovibrio
Vibrionaceae;Vibrio
Sporolactobacillaceae;Sporolactobacillus
Comamonadaceae;Aquabacterium
Bryobacteraceae;Bryobacter
Pseudoalteromonadaceae;Pseudoalteromonas
Beijerinckiaceae;Roseiarcus
Moraxellaceae;Psychrobacter
Flavobacteriaceae;Lutimonas
Staphylococcaceae;Staphylococcus
Hydrogenophilaceae;Tepidiphilus
Peptostreptococcaceae;Sporacetigenium
Intrasporangiaceae;Arsenicicoccus
Comamonadaceae;Pelomonas
Flavobacteriaceae;Tenacibaculum
Burkholderiaceae;Polynucleobacter
−2 0 2 4
0 5 10 15 20
−200 −100 0 100
−200 −100 0 100 200
Covariates

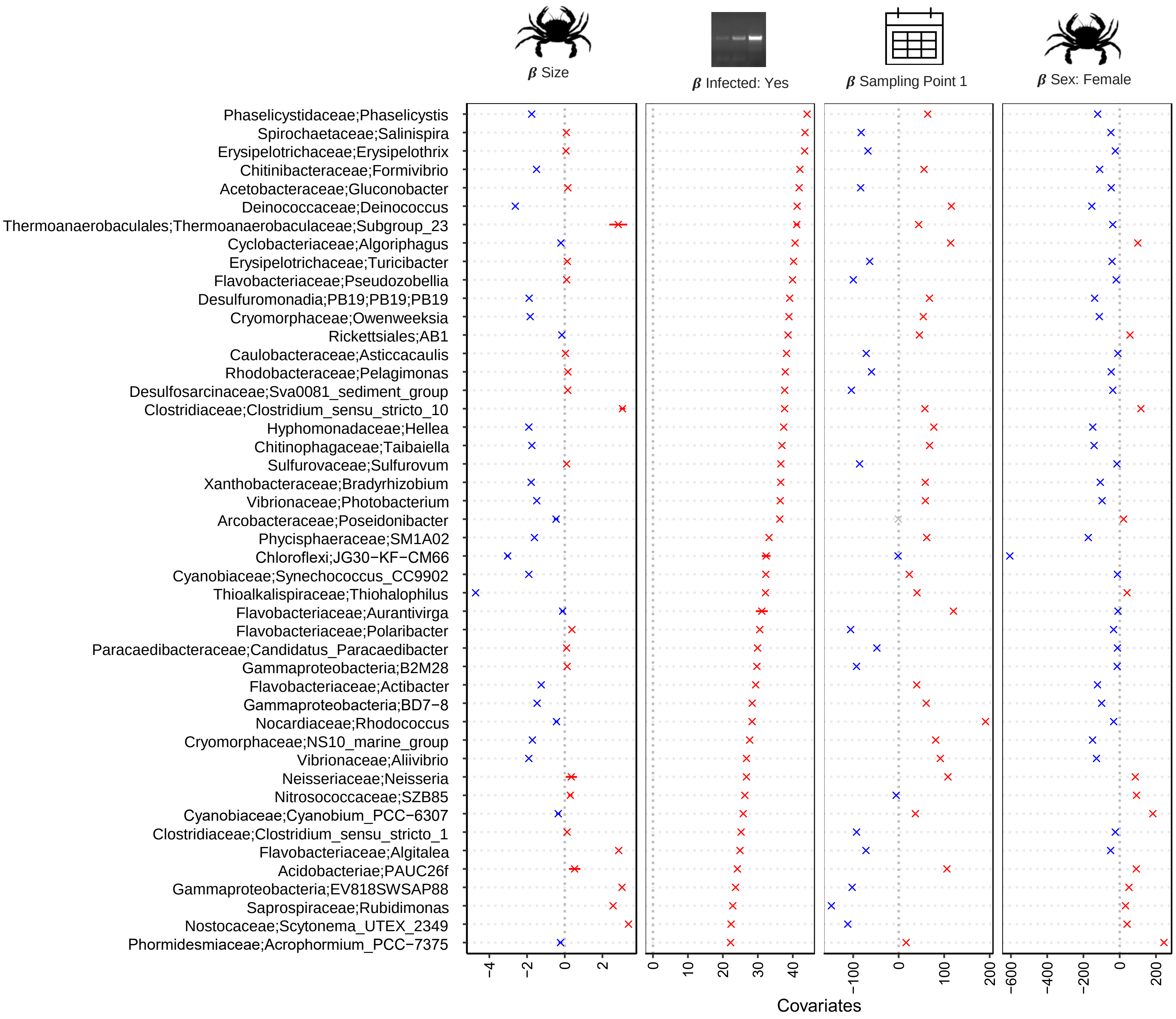
β Size
β Infected: Yes
β Sampling Point 1
β Sex: Female
Phaselicystidaceae;Phaselicystis
Spirochaetaceae;Salinispira
Erysipelotrichaceae;Erysipelothrix
Chitinibacteraceae;Formivibrio
Acetobacteraceae;Gluconobacter
Deinococcaceae;Deinococcus
Thermoanaerobaculales;Thermoanaerobaculaceae;Subgroup_23
Cyclobacteriaceae;Algoriphagus
Erysipelotrichaceae;Turicibacter
Flavobacteriaceae;Pseudozobellia
Desulfuromonadia;PB19;PB19;PB19
Cryomorphaceae;Owenweeksia
Rickettsiales;AB1
Caulobacteraceae;Asticcacaulis
Rhodobacteraceae;Pelagimonas
Desulfosarcinaceae;Sva0081_sediment_group
Clostridiaceae;Clostridium_sensu_stricto_10
Hyphomonadaceae;Hellea
Chitinophagaceae;Taibaiella
Sulfurovaceae;Sulfurovum
Xanthobacteraceae;Bradyrhizobium
Vibrionaceae;Photobacterium
Arcobacteraceae;Poseidonibacter
Phycisphaeraceae;SM1A02
Chloroflexi;JG30−KF−CM66
Cyanobiaceae;Synechococcus_CC9902
Thioalkalispiraceae;Thiohalophilus
Flavobacteriaceae;Aurantivirga
Flavobacteriaceae;Polaribacter
Paracaedibacteraceae;Candidatus_Paracaedibacter
Gammaproteobacteria;B2M28
Flavobacteriaceae;Actibacter
Gammaproteobacteria;BD7−8
Nocardiaceae;Rhodococcus
Cryomorphaceae;NS10_marine_group
Vibrionaceae;Aliivibrio
Neisseriaceae;Neisseria
Nitrosococcaceae;SZB85
Cyanobiaceae;Cyanobium_PCC−6307
Clostridiaceae;Clostridium_sensu_stricto_1
Flavobacteriaceae;Algitalea
Acidobacteriae;PAUC26f
Gammaproteobacteria;EV818SWSAP88
Saprospiraceae;Rubidimonas
Nostocaceae;Scytonema_UTEX_2349
Phormidesmiaceae;Acrophormium_PCC−7375
−4
−2
0
2
0
10
20
30
40
−100
0
100
200
−600
−400
−200
0
200
Covariates

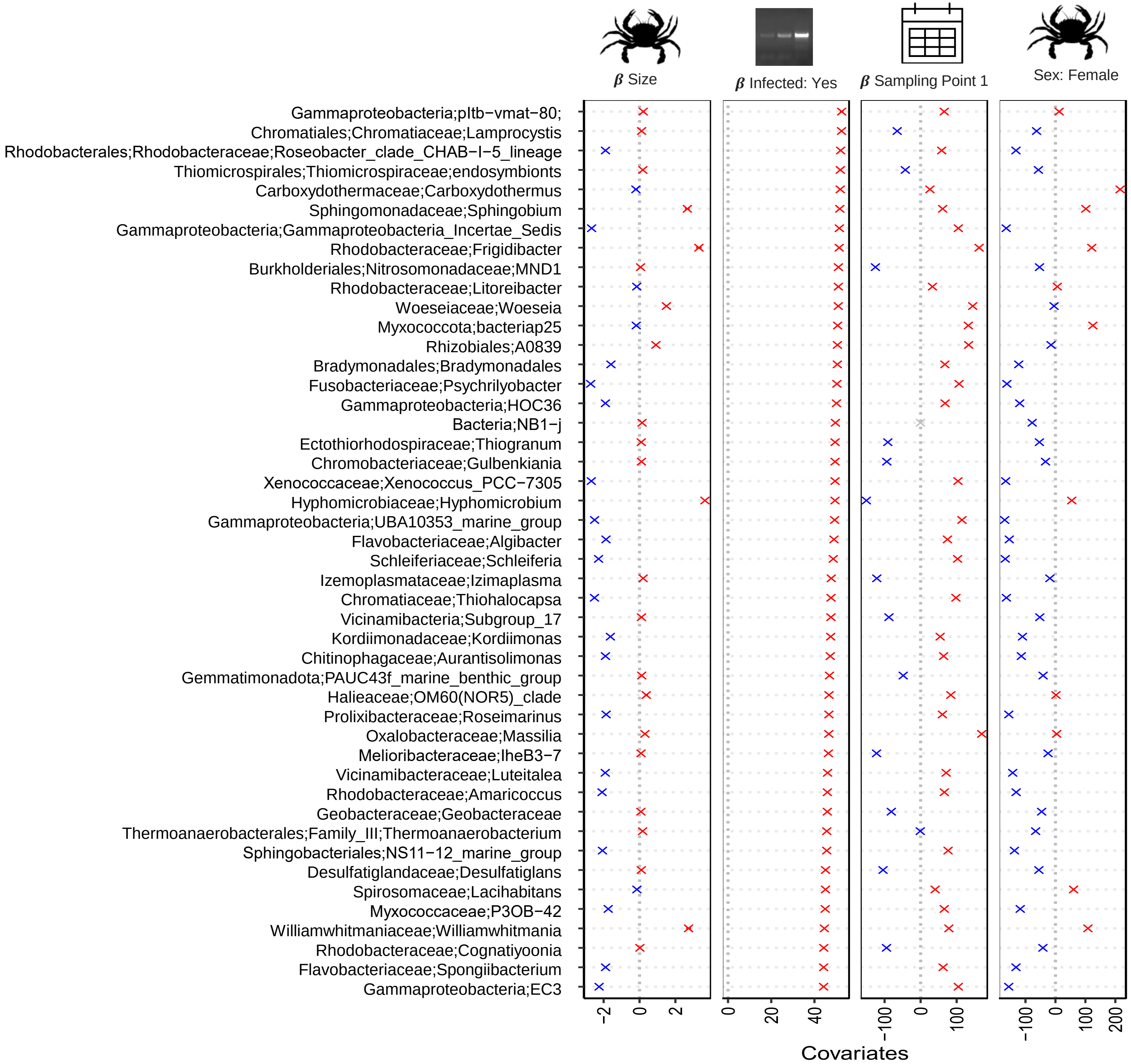
β Size
β Infected: Yes
β Sampling Point 1
Sex: Female
Gammaproteobacteria;pltb−vmat−80;
Chromatiales;Chromatiaceae;Lamprocystis
Rhodobacterales;Rhodobacteraceae;Roseobacter_clade_CHAB−I−5_lineage
Thiomicrospirales;Thiomicrospiraceae;endosymbionts
Carboxydothermaceae;Carboxydothermus
Sphingomonadaceae;Sphingobium
Gammaproteobacteria;Gammaproteobacteria_Incertae_Sedis
Rhodobacteraceae;Frigidibacter
Burkholderiales;Nitrosomonadaceae;MND1
Rhodobacteraceae;Litoreibacter
Woeseiaceae;Woeseia
Myxococcota;bacteriap25
Rhizobiales;A0839
Bradymonadales;Bradymonadales
Fusobacteriaceae;Psychrilyobacter
Gammaproteobacteria;HOC36
Bacteria;NB1−j
Ectothiorhodospiraceae;Thiogranum
Chromobacteriaceae;Gulbenkiania
Xenococcaceae;Xenococcus_PCC−7305
Hyphomicrobiaceae;Hyphomicrobium
Gammaproteobacteria;UBA10353_marine_group
Flavobacteriaceae;Algibacter
Schleiferiaceae;Schleiferia
Izemoplasmataceae;Izimaplasma
Chromatiaceae;Thiohalocapsa
Vicinamibacteria;Subgroup_17
Kordiimonadaceae;Kordiimonas
Chitinophagaceae;Aurantisolimonas
Gemmatimonadota;PAUC43f_marine_benthic_group
Halieaceae;OM60(NOR5)_clade
Prolixibacteraceae;Roseimarinus
Oxalobacteraceae;Massilia
Melioribacteraceae;IheB3−7
Vicinamibacteraceae;Luteitalea
Rhodobacteraceae;Amaricoccus
Geobacteraceae;Geobacteraceae
Thermoanaerobacterales;Family_III;Thermoanaerobacterium
Sphingobacteriales;NS11−12_marine_group
Desulfatiglandaceae;Desulfatiglans
Spirosomaceae;Lacihabitans
Myxococcaceae;P3OB−42
Williamwhitmaniaceae;Williamwhitmania
Rhodobacteraceae;Cognatiyoonia
Flavobacteriaceae;Spongiibacterium
Gammaproteobacteria;EC3
−2
0
2
0
20
40
−100
0
100
−100
0
100
200
Covariates

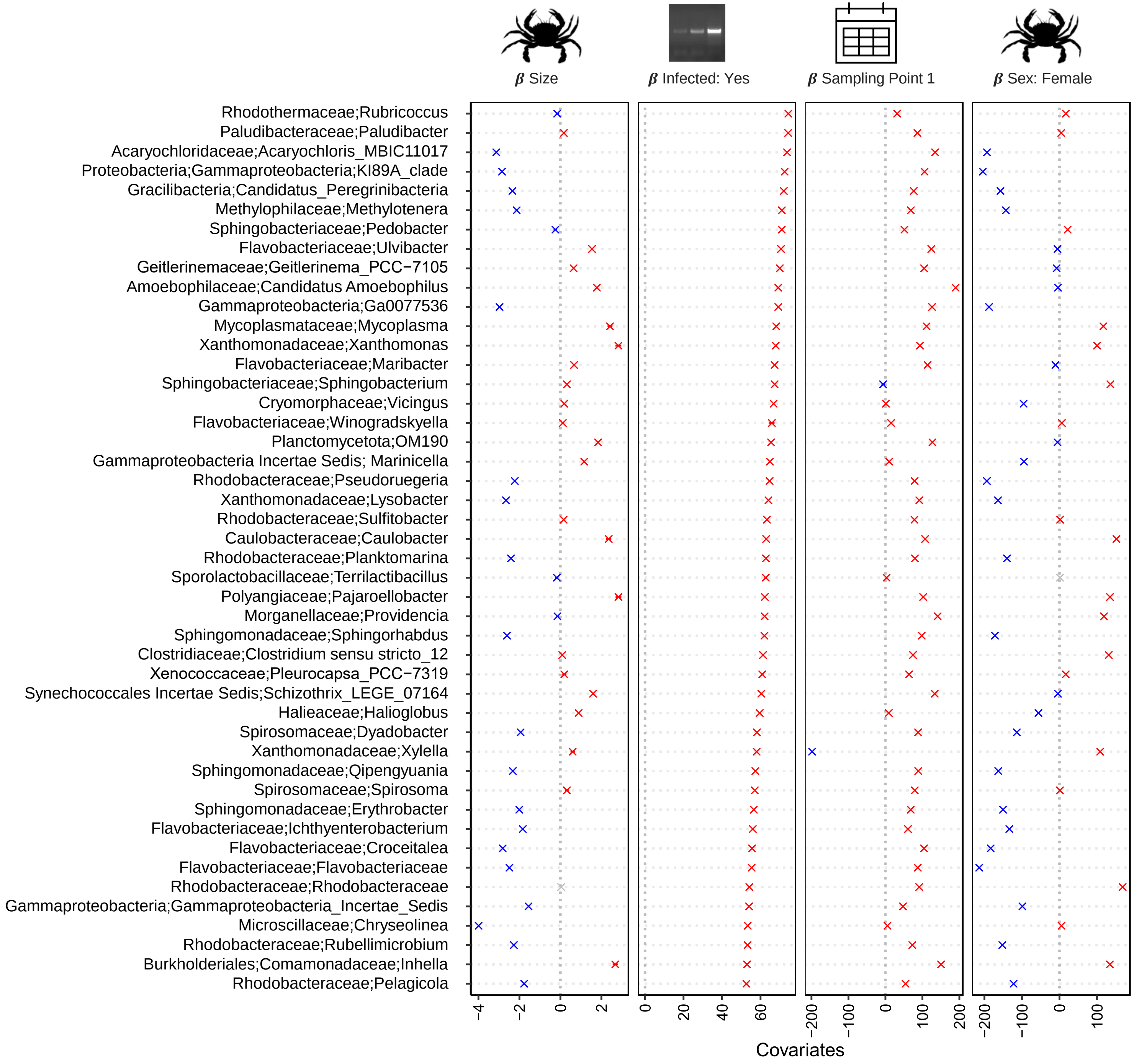

β Size
β Infected: Yes
β Sampling Point 1
β Sex: Female
Rhodothermaceae;Rubricoccus
Paludibacteraceae;Paludibacter
Acaryochloridaceae;Acaryochloris_MBIC11017
Proteobacteria;Gammaproteobacteria;KI89A_clade
Gracilibacteria;Candidatus_Peregrinibacteria
Methylophilaceae;Methylotenera
Sphingobacteriaceae;Pedobacter
Flavobacteriaceae;Ulvibacter
Geitlerinemaceae;Geitlerinema_PCC−7105
Amoebophilaceae;Candidatus Amoebophilus
Gammaproteobacteria;Ga0077536
Mycoplasmataceae;Mycoplasma
Xanthomonadaceae;Xanthomonas
Flavobacteriaceae;Maribacter
Sphingobacteriaceae;Sphingobacterium
Cryomorphaceae;Vicingus
Flavobacteriaceae;Winogradskyella
Planctomycetota;OM190
Gammaproteobacteria Incertae Sedis; Marinicella
Rhodobacteraceae;Pseudoruegeria
Xanthomonadaceae;Lysobacter
Rhodobacteraceae;Sulfitobacter
Caulobacteraceae;Caulobacter
Rhodobacteraceae;Planktomarina
Sporolactobacillaceae;Terrilactibacillus
Polyangiaceae;Pajaroellobacter
Morganellaceae;Providencia
Sphingomonadaceae;Sphingorhabdus
Clostridiaceae;Clostridium sensu stricto_12
Xenococcaceae;Pleurocapsa_PCC−7319
Synechococcales Incertae Sedis;Schizothrix_LEGE_07164
Halieaceae;Halioglobus
Spirosomaceae;Dyadobacter
Xanthomonadaceae;Xylella
Sphingomonadaceae;Qipengyuania
Spirosomaceae;Spirosoma
Sphingomonadaceae;Erythrobacter
Flavobacteriaceae;Ichthyenterobacterium
Flavobacteriaceae;Croceitalea
Flavobacteriaceae;Flavobacteriaceae
Rhodobacteraceae;Rhodobacteraceae
Gammaproteobacteria;Gammaproteobacteria_Incertae_Sedis
Microscillaceae;Chryseolinea
Rhodobacteraceae;Rubellimicrobium
Burkholderiales;Comamonadaceae;Inhella
Rhodobacteraceae;Pelagicola
−4 −2 0 2
0 20 40 60
−200 −100 0 100 200
−200 −100 0 100
Covariates

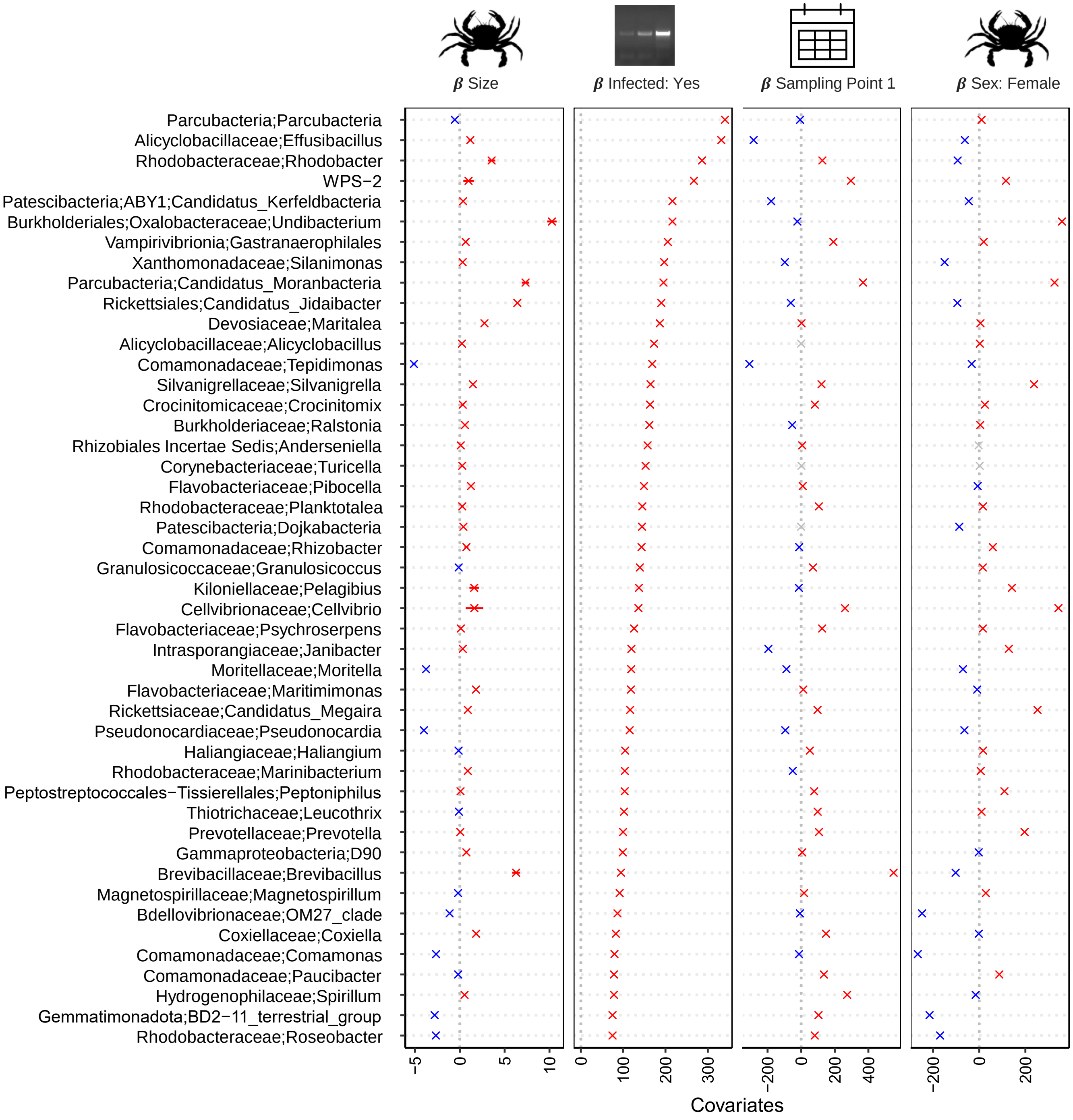

β Size
β Infected: Yes
β Sampling Point 1
β Sex: Female
Parcubacteria;Parcubacteria
Alicyclobacillaceae;Effusibacillus
Rhodobacteraceae;Rhodobacter
WPS−2
Patescibacteria;ABY1;Candidatus_Kerfeldbacteria
Burkholderiales;Oxalobacteraceae;Undibacterium
Vampirivibrionia;Gastranaerophilales
Xanthomonadaceae;Silanimonas
Parcubacteria;Candidatus_Moranbacteria
Rickettsiales;Candidatus_Jidaibacter
Devosiaceae;Maritalea
Alicyclobacillaceae;Alicyclobacillus
Comamonadaceae;Tepidimonas
Silvanigrellaceae;Silvanigrella
Crocinitomicaceae;Crocinitomix
Burkholderiaceae;Ralstonia
Rhizobiales Incertae Sedis;Anderseniella
Corynebacteriaceae;Turicella
Flavobacteriaceae;Pibocella
Rhodobacteraceae;Planktotalea
Patescibacteria;Dojkabacteria
Comamonadaceae;Rhizobacter
Granulosicoccaceae;Granulosicoccus
Kiloniellaceae;Pelagibius
Cellvibrionaceae;Cellvibrio
Flavobacteriaceae;Psychroserpens
Intrasporangiaceae;Janibacter
Moritellaceae;Moritella
Flavobacteriaceae;Maritimimonas
Rickettsiaceae;Candidatus_Megaira
Pseudonocardiaceae;Pseudonocardia
Haliangiaceae;Haliangium
Rhodobacteraceae;Marinibacterium
Peptostreptococcales−Tissierellales;Peptoniphilus
Thiotrichaceae;Leucothrix
Prevotellaceae;Prevotella
Gammaproteobacteria;D90
Brevibacillaceae;Brevibacillus
Magnetospirillaceae;Magnetospirillum
Bdellovibrionaceae;OM27_clade
Coxiellaceae;Coxiella
Comamonadaceae;Comamonas
Comamonadaceae;Paucibacter
Hydrogenophilaceae;Spirillum
Gemmatimonadota;BD2−11_terrestrial_group
Rhodobacteraceae;Roseobacter
−5
0
5
10
0
100
200
300
−200
0
200
400
−200
0
200
Covariates
