# Supplementary material for: The hepatopancreas microbiome of velvet crab, Necora puber
Source: Environ Microbiol Rep. 2024 Oct 1;16(5):e70014. doi: 10.1111/1758-2229.70014 (PMC11445078; doi:10.1111/1758-2229.70014)
Supplement: Supplementary file 3 — Figure S4. (i) Taxonomic coverage tree of the core velvet crab hepatopancreas microbiome showing collated abundances across all occupancies (sampling point [1/2], infection with Paramarteilia canceri [Yes/No], and sex [Male/Female]). The key on the right side of the tree can be interpreted as follows: the width of the bar represents the number of unique taxa and is the size of the nodes (shown on the left side of the key), while the colour represents the count of these taxa (shown on the right side of the key). (ii) Taxonomic coverage tree of the core velvet crab hepatopancreas microbiome for the occupancy; Sampling point 1; Not infected; Female. Interpretation is the same as what is mentioned in the legend of Figure S4, i. (iii) Taxonomic coverage tree of the core velvet crab hepatopancreas microbiome for the occupancy; Sampling point 1; Not infected; Male. Interpretation is the same as what is mentioned in the legend of Figure S4, i. (iv) Taxonomic coverage tree of the core velvet crab hepatopancreas microbiome for the occupancy; Sampling point 1; Infected; Female. Interpretation is the same as what is mentioned in the legend of Figure S4, i. (v) Taxonomic coverage tree of the core velvet crab hepatopancreas microbiome for the occupancy; Sampling point 1; Infected; Male. Interpretation is the same as what is mentioned in the legend of Figure S4, i. (vi) Taxonomic coverage tree of the core velvet crab hepatopancreas microbiome for the occupancy; Sampling point 2; Not infected; Female. Interpretation is the same as what is mentioned in the legend of Figure S4, i. (vii) Taxonomic coverage tree of the core velvet crab hepatopancreas microbiome for the occupancy; Sampling point 2; Infected; Female. Interpretation is the same as what is mentioned in the legend of Figure S4, i. (viii) Taxonomic coverage tree of the core velvet crab hepatopancreas microbiome for the occupancy; Sampling point 2; Infected; Male. Interpretation is the same as what is mentioned in the legend [file EMI4-16-e70014-s002.pdf]

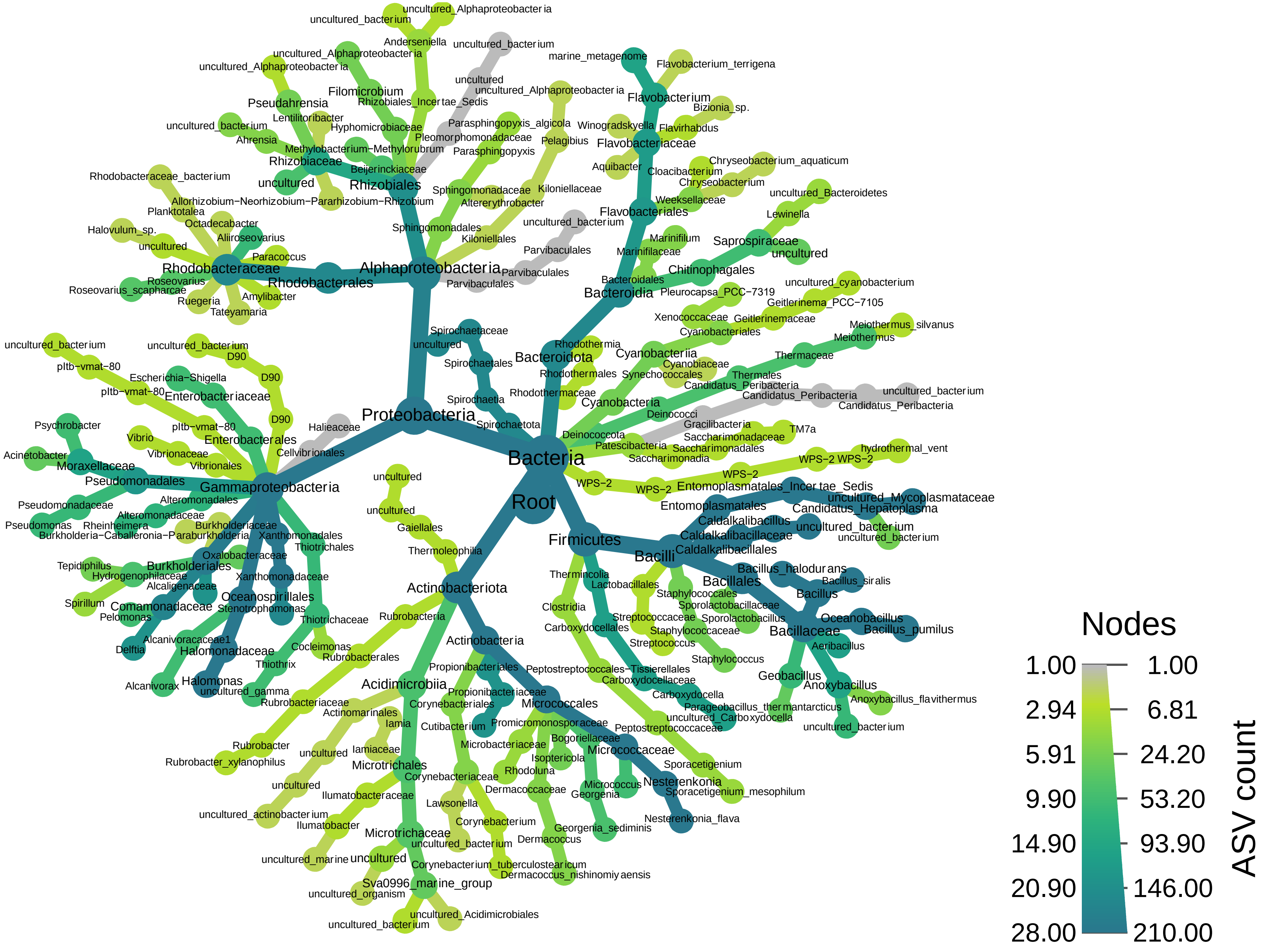

Core: All treatment-specific occupancies collated together

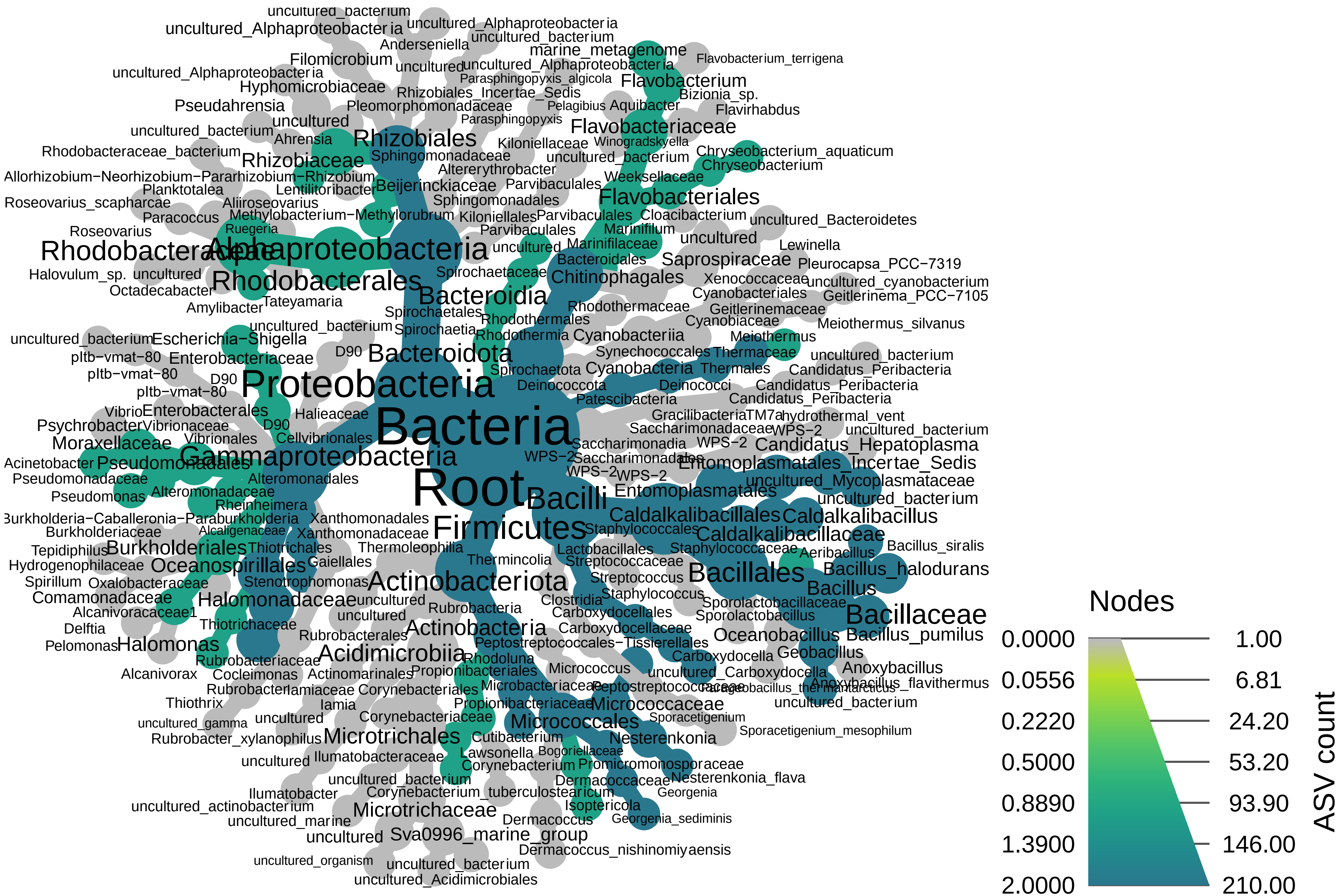

Core at occupancy: Sampling point 1; Not infected; Female

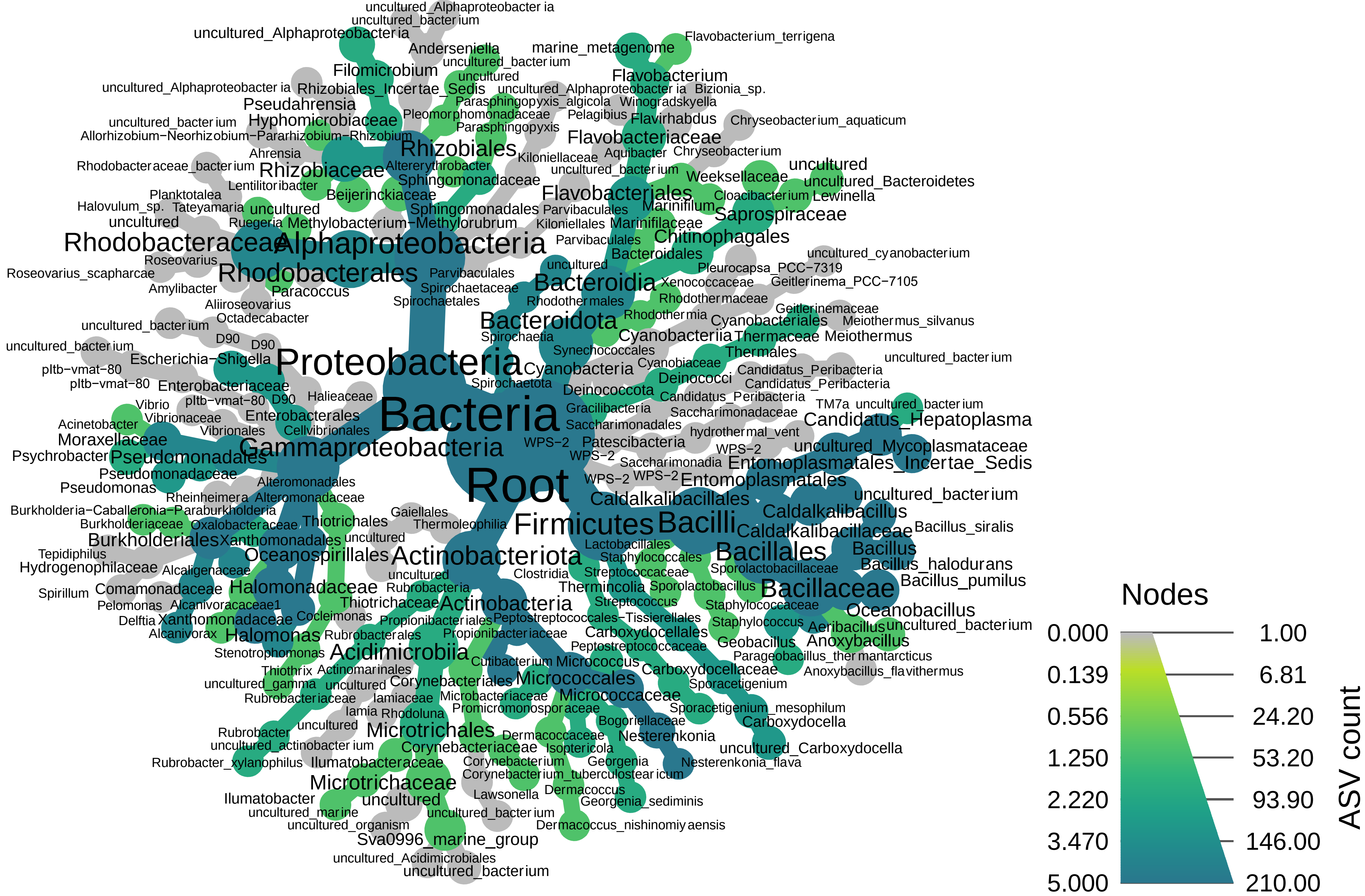

Core at occupancy: Sampling point 1; Not infected; Male

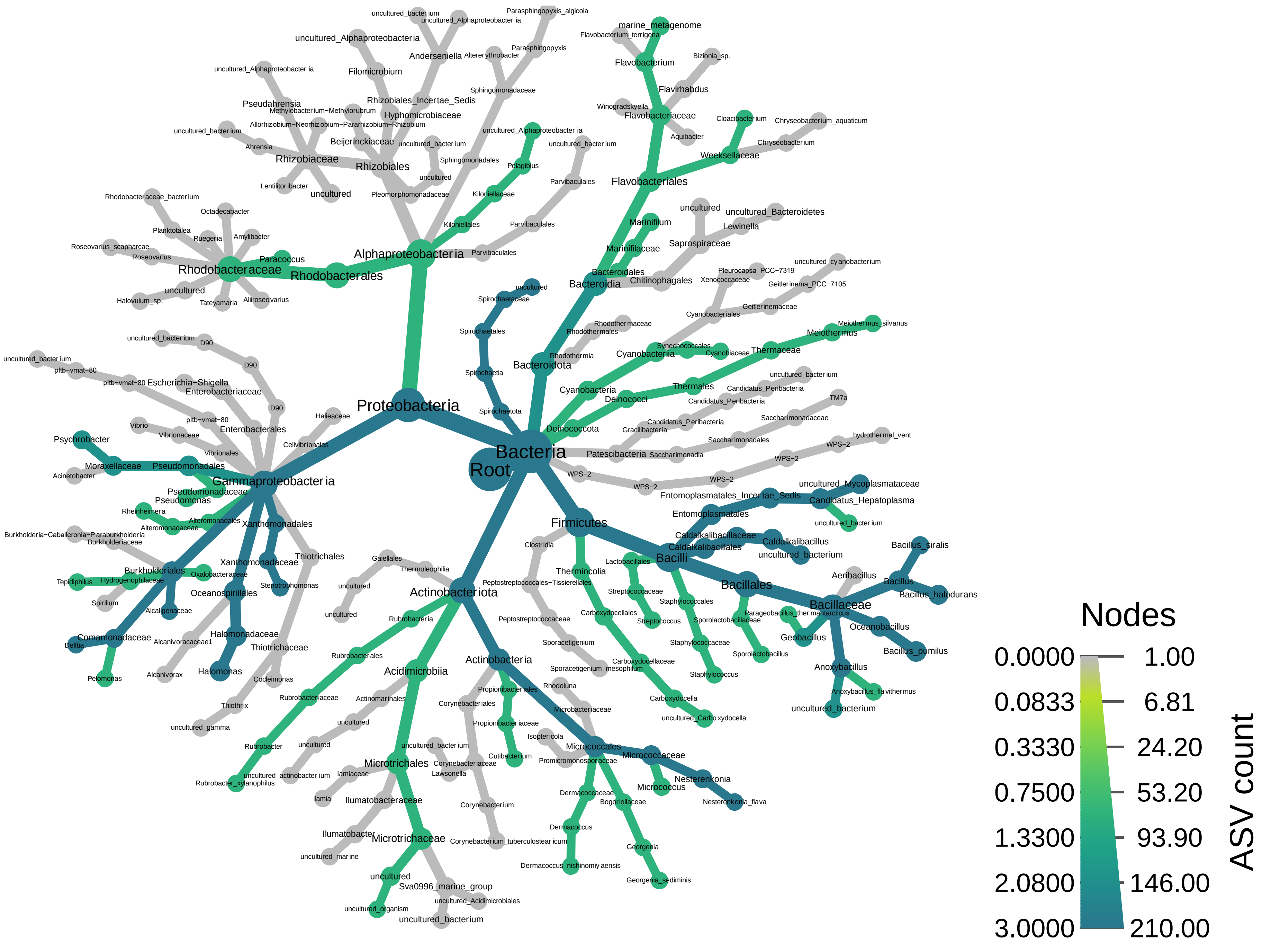

Core at occupancy: Sampling point 1; Infected; Female

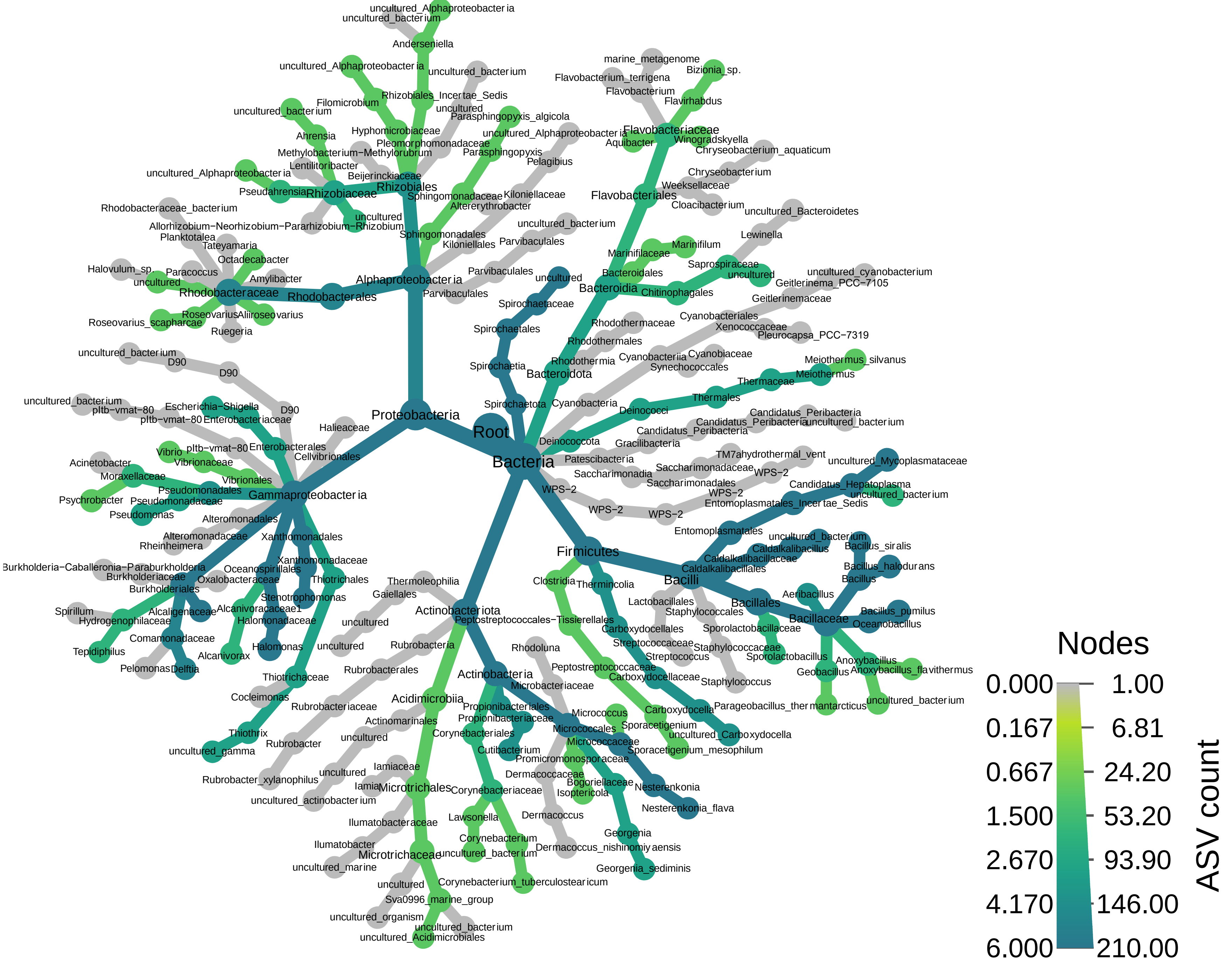

Core at occupancy: Sampling point 1; Infected; Male

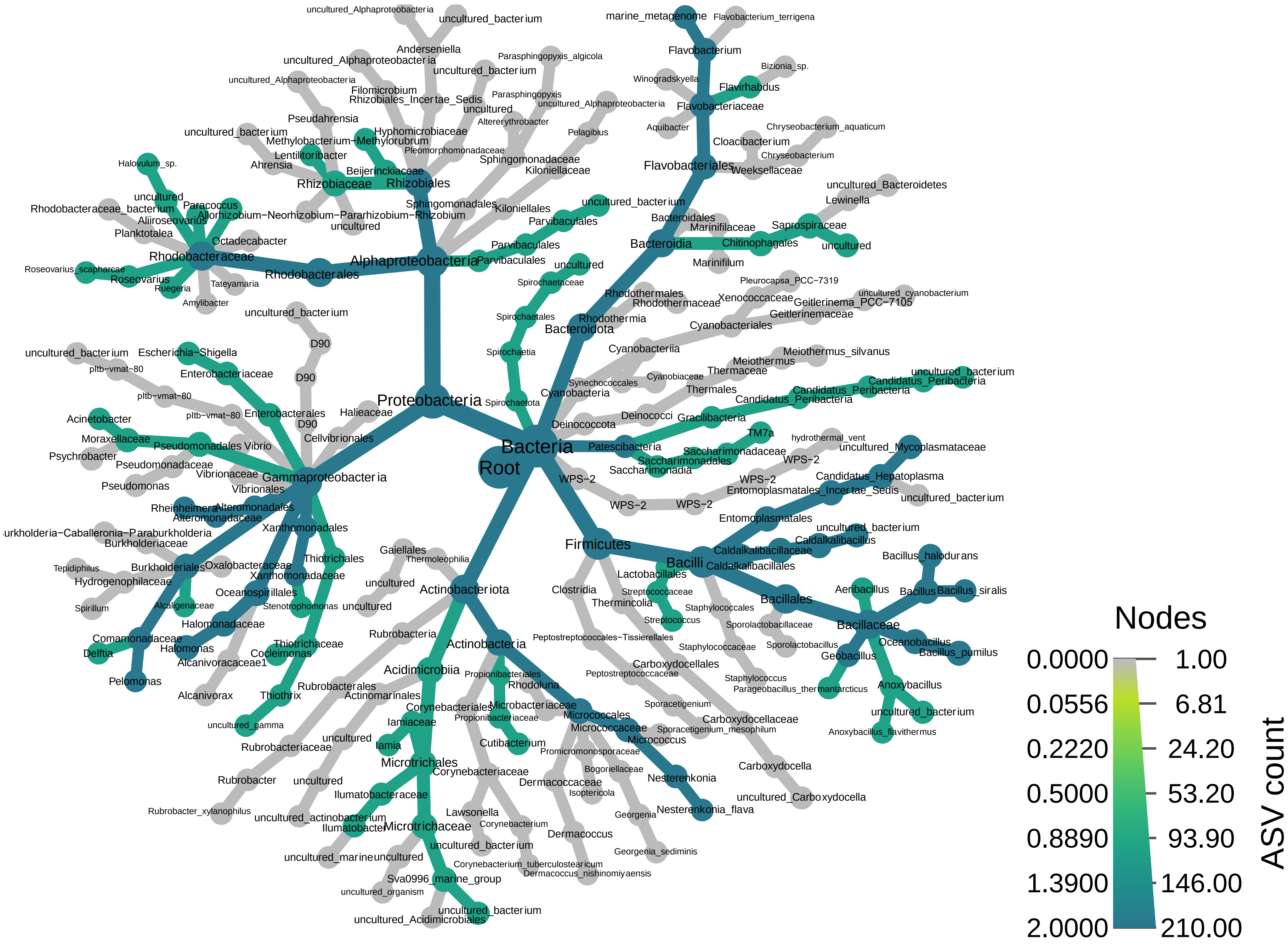

Core at occupancy: Sampling point 2; Not infected; Female

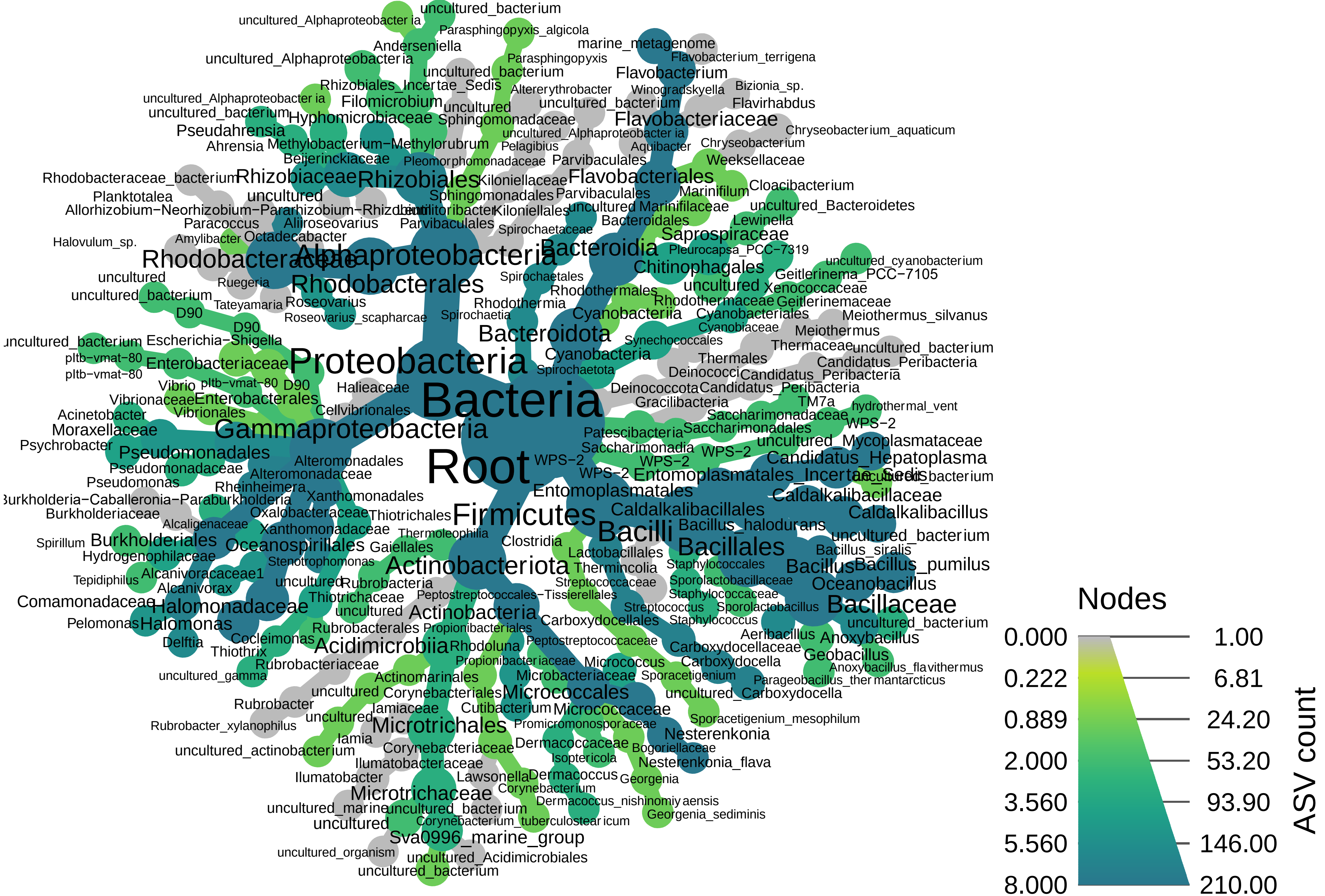

Core at occupancy: Sampling point 2; Infected; Female

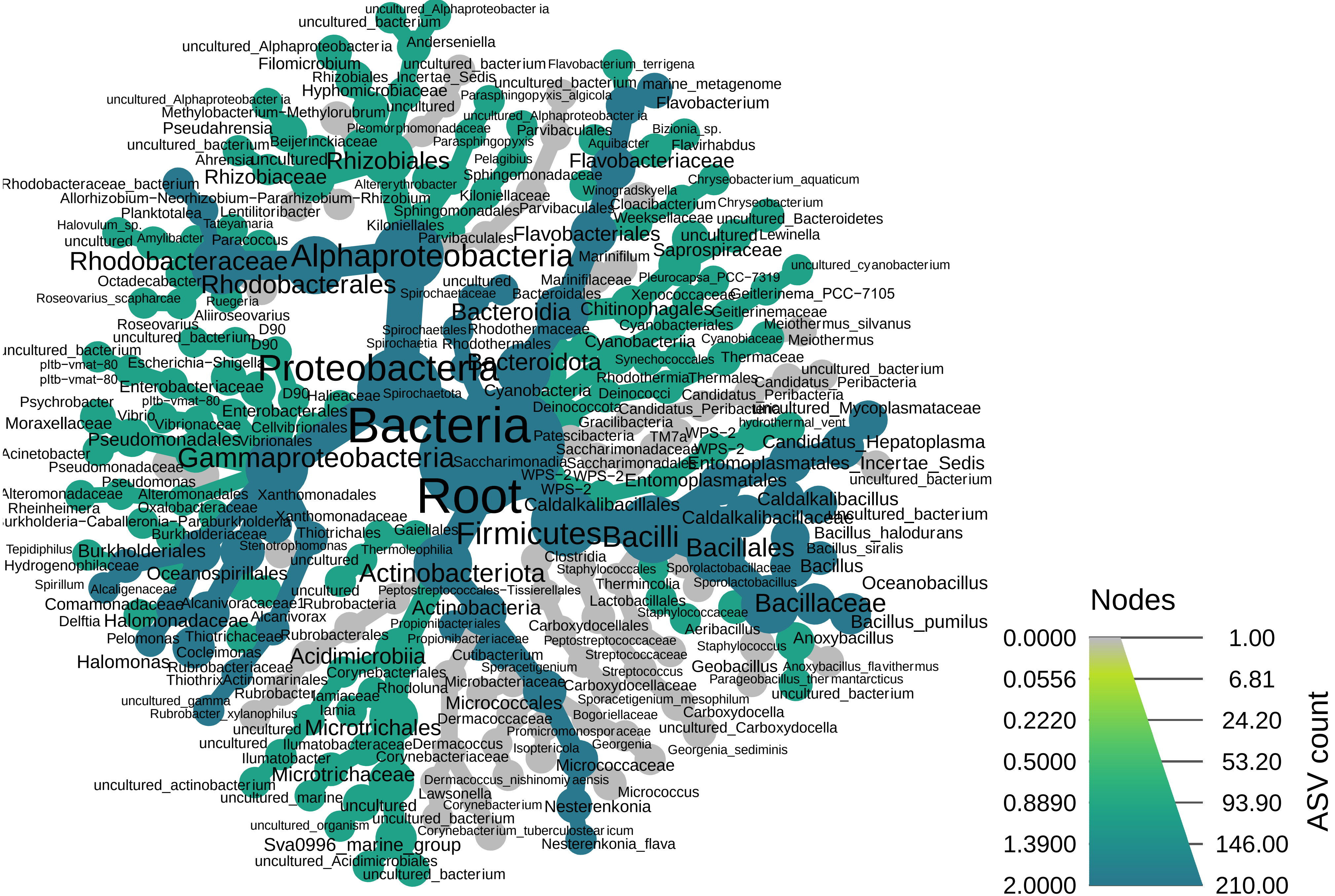

Core at occupancy: Sampling point 2; Infected; Male
